# Transcriptional and metabolic profiling of sulfur starvation response in two monocots

**DOI:** 10.1186/s12870-024-04948-2

**Published:** 2024-04-09

**Authors:** Ivan Zenzen, Daniela Cassol, Philipp Westhoff, Stanislav Kopriva, Daniela Ristova

**Affiliations:** 1 Institute for Plant Sciences, Cluster of Excellence On Plant Sciences (CEPLAS), University of Cologne, Cologne, 50674 Germany; 2 Institute for Integrative Genome Biology, University of California, Riverside, 92521 CA USA; 3 Plant Metabolism and Metabolomics Facility, Heinrich Heine University, Düsseldorf, 40225 Germany

**Keywords:** Sulfur metabolism, Sulfate deficiency, Plant nutrition, Rice, *Setaria viridis*, Transcriptomics, Metabolomics

## Abstract

**Background:**

Sulfur (S) is a mineral nutrient essential for plant growth and development, which is incorporated into diverse molecules fundamental for primary and secondary metabolism, plant defense, signaling, and maintaining cellular homeostasis. Although, S starvation response is well documented in the dicot model *Arabidopsis thaliana*, it is not clear if the same transcriptional networks control the response also in the monocots.

**Results:**

We performed series of physiological, expression, and metabolite analyses in two model monocot species, one representing the C_3_ plants, *Oryza sativa* cv. *kitaake*, and second representing the C_4_ plants, *Setaria viridis*. Our comprehensive transcriptomic analysis revealed twice as many differentially expressed genes (DEGs) in *S. viridis* than in *O. sativa* under S-deficiency, consistent with a greater loss of sulfur and S-containing metabolites under these conditions. Surprisingly, most of the DEGs and enriched gene ontology terms were species-specific, with an intersect of only 58 common DEGs. The transcriptional networks were different in roots and shoots of both species, in particular no genes were down-regulated by S-deficiency in the roots of both species.

**Conclusions:**

Our analysis shows that S-deficiency seems to have different physiological consequences in the two monocot species and their nutrient homeostasis might be under distinct control mechanisms.

**Supplementary Information:**

The online version contains supplementary material available at 10.1186/s12870-024-04948-2.

## Background

Sulfur (S) is an essential nutrient for plant growth and development. Plants incorporate S in various important molecules, including amino acids, proteins, enzymes, vitamins, and coenzymes. In protein synthesis, sulfur plays a central role as a component of the amino acids cysteine (Cys) and methionine (Met), which are building blocks for enzymes, structural proteins, and other proteins that ultimately shape plant metabolism [1]. S is also a component of important plant defense compounds, such as glucosinolates, that protect the plant against herbivores and pests and confer the pungent taste and smell of plants in the Brassicaceae, with potential health benefits for humans [2, 3]. Sulfur is also necessary for the biosynthesis of plant hormones, including ethylene, and abscisic acid, which for instance, play an important function in the regulation of plant growth and development being involved in the control of plant senescence, seed germination, and drought tolerance [4]. Additionally, an often overlooked aspect of sulfur nutrition in plants is the important role this macronutrient plays in facilitating uptake and utilization of other nutrients. Besides helping to maintain the proper pH level in the soil, which affects the availability of other nutrients, such as phosphorus and micronutrients, sulfur also interacts in a more direct manner with core metabolic processes such as carbon and nitrogen assimilation pathways [5–7].

Research on S signaling and starvation response in crops is lagging, compared to other macronutrients, mostly because S deficiency in modern agriculture was not an issue until recently [8]. However, due to the implementation of 1990 Clean Air Act Amendments and significant reduction of atmospheric S emissions [9], S deficiency is becoming a threat to modern agriculture practice, especially when combined with other deficiencies, because the combined effects are not well understood at physiological level [7]. Long-term S starvation response is well characterized in *Arabidopsis thaliana*, at transcriptional and physiological levels [10–12]. Growth retardation and chlorosis of S deprived plants was noticed about seven to ten days after S deprivation [12]. At metabolic level, under S starvation plants are sustaining their growth by using of all possible sulfate from the environment and internally, by increasing sulfate uptake, releasing vacuolar sulfate, and catabolic recycling of secondary sulfur metabolites [10–13]. Therefore, shoots of S starved plants had significant decrease of total S, sulfate, Met, glutathione (GSH) [11, 12], aliphatic and indolic glucosinolates, and increased sulfate uptake and translocation rate [10]. However, several fold accumulation of O-acetyl-serine (OAS) was observed, which together with reduced sulfide forms Cys, as well as multiple fold increase in tryptophan [12].

*ETHYLENE-INSENSITIVE3-LIKE3 (EIL3)* or *SULFUR LIMITATION1 (SLIM1)* was the first transcriptional factor identified as main regulator of multiple genes, involved in both the activation of sulfate acquisition and degradation of glucosinolates under S starvation conditions [11]. However, SLIM1/EIL3 expression was not altered under S limitation, suggesting post-transcriptional regulation [10, 11]. Sulfate uptake was about 60% reduced in the *slim1* mutant exposed to S-deficiency, as well as transcript levels of several sulfate transporters, such as *SULTR1;1, SULTR1;2, SULTR3;4*, and *SULTR4;2* [10, 11]. Another obvious difference in the transcriptome of *slim1* mutant was lower degree of downregulation of genes for glucosinolate synthesis, and attenuated upregulation of glucosinolate catabolism [10, 11]. Recent comparison of both datasets, revealed a core set of 38 genes regulated by SLIM1/EIL3 under S starvation in *Arabidopsis thaliana* [8] divided in two main clusters. The larger cluster, contains genes that are highly upregulated under S starvation in wild-type plants, but are significantly less induced in the *slim1/eil3* mutant, including genes for sulfate transporters (*SULTR1;1, SULTR1;2, SULTR4;2*), sulfate assimilation (*SERAT3;1*), negative regulation of glucosinolate biosynthesis (*SDI1*), epigenetic regulation of sulfur homeostasis (*SHM7*), *LSU1* and *LSU2* involved in response to low S, and cellular detoxification and defense response, respectively, and another 20 genes that are dependent on SLIM1/EIL3, suggesting that *EIL3/SLIM1* is orchestrating complex physiological changes in the plant under S limitation [8].

The knowledge on regulation of S metabolism and response to S starvation beyond Arabidopsis is rather patchy, but there is clear evidence for species/lineages specific mechanisms [14]. One of the major alterations is the different spatial organization of sulfate assimilation in C_3_ and C_4_ monocot plants [15–17]. C_4_ photosynthesis has crucial impact on numerous pathways and metabolites because of the spatial separation between mesophyll cells (MCs) and bundle sheath cells (BSCs) where all cellular processes need to be adjusted, including nitrogen (N) and S assimilation [18]. Higher photosynthetic efficiency in C_4_ compared to C_3_ plants is achieved by strong reduction of the oxygenase reaction of Rubisco and photorespiration, leading to increased biomass production in crops, which in turn requires enhanced mineral nutrient acquisition, and specific adaptations in N and S metabolism [15–17]. However, S assimilation is restricted to BSCs only in C_4_ monocots [7, 15–17, 19], whereas in the C_4_ dicots the pathway is found in both cell types. Interestingly, a gradual increase of foliar accumulation of reduced sulfur compounds Cys and GSH from C_3_ to C_4_* Flaveria* species was observed [16, 17]. Thus, not only is there a difference between plants with C_3_ and C_4_ photosynthesis but also between dicots and monocots. In addition, recently it was shown that two monocot model species *Oryza sativa* and *Setaria viridis* are capable of oxidizing cysteine to sulfate, a reaction that is not present in Arabidopsis [20]. These findings suggest that during plant evolution sulfur metabolism underwent distinct ranges of adaptation in different lineages and, therefore, detailed studies are needed to understand how molecular networks controlling S assimilation are adapted in different species, which components are common and which are species specific.

In order to identify such different regulatory networks, in this study, we analyzed global transcriptional response to S deficiency in two monocots species, with two photosynthetic types C_3_ and C_4_. We identified small number of overlapping DEGs, and most of the enriched GO terms were species-specific. One common transcriptional signature was degradation of Met, which seems to be crucial for coping with S starvation in multiple species. Network analysis pinpointed two genes involved in phosphate transport and signaling as novel candidates contributing to S starvation response. We further identified metabolic signature of higher accumulation of branched chain amino acids specifically in *S. viridis*, but not *O. sativa*. Our findings suggest that S-deficiency probably has different physiological consequences in different species and nutrient homeostasis might be under distinct control mechanisms.

## Results

### Sulfur deficiency phenotypes in *O. sativa* and *S. viridis*

**Fig. 1:**
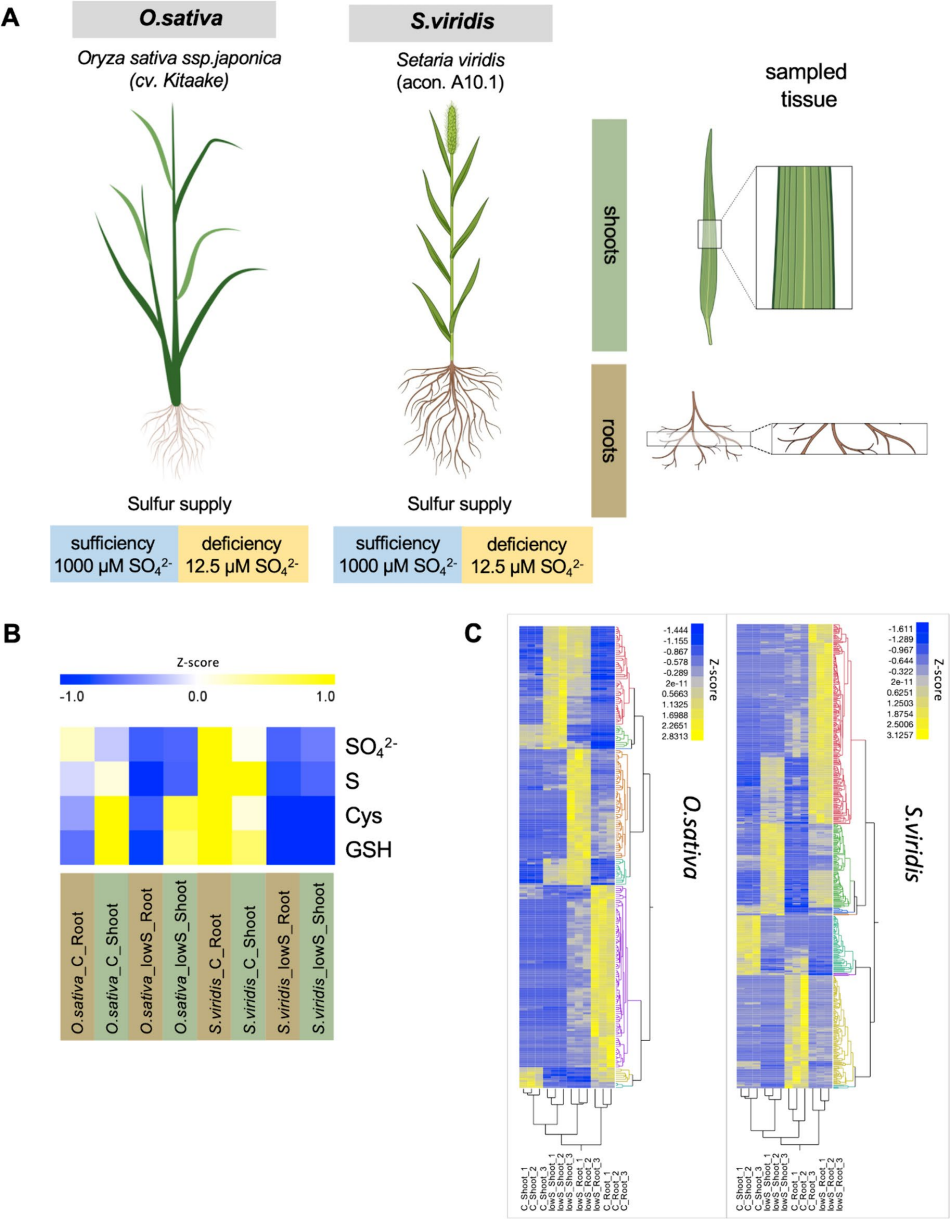
S deficiency response in two monocots: *O. sativa* and *S. viridis*, representing C_3_ and C_4_ photosynthesis types. **A** Diagram of the experimental design (created in Biorender). Plants were grown hydroponically for 27 days, and shoot and root tissues in the middle section was sampled for analysis. In total 24 samples were used to perform RNA-Seq analysis (2 species* 2 conditions* 2 organs* 3 replicates). **B** Quantification of elemental S, sulfate and the thiols cysteine (Cys) and glutathione (GSH) under S deficiency. **C** Hierarchical clustering (average) on differentially expressed genes (DEGs) in C_3_, *O. sativa* and C_4_, *S. viridis.* Full media corresponds to 1000 µM SO_4_^2−^ (**C**), and S deficiency corresponds to 12.5 µM SO_4_^2−^ (lowS)

After 20 days of growth under sulfur deficiency treatment (12.5 µM SO_4_^2−^), plants of *O. sativa* showed a decrease of about 49% in total biomass accumulation, while in *S. viridis*, the reduction in growth represented about 67%, compared to their respective controls (Supplemental Fig. 1A). The decrease in biomass accumulation was accompanied by a sharp decrease in the organic and inorganic sulfur pools in the plant tissues, with total sulfur decreased by approximately 73% and 83% in roots of *O. sativa* and *S. viridis*, respectively, with a relative reduction of 56% and 71% in shoots of both species (Supplemental Fig. 1B). Sulfate, a common inorganic sulfur storage in plant cells, dropped by approximately 65% in shoots of both species, accompanied by a decrease by 71% in roots of *O. sativa,* and 84% decrease in the below ground tissues of *S. viridis* (Fig. 1A,B, Supplemental Fig. 1C). Regarding the reduced sulfur present in organic molecules, cysteine, which represents the first stable organic sulfur compound from primary sulfur assimilation pathway, did not show significant changes upon sulfur deficiency in tissues of *O. sativa*, although a sharp decrease was observed in both root and shoot tissues from *S. viridis*, corresponding to approximately 87% and 79% lower levels relative to control plants, respectively (Fig. 1B, Supplemental Fig. 1D). While sulfur deficiency caused a relative reduction of 40% and 30% in glutathione content in *O. sativa* roots and shoots, respectively, a staggering decrease by over 97% and 95% was noticed in the corresponding tissues from *S. viridis* plants (Fig. 1B, Supplemental Fig. 1E).

### Generation of high-resolution transcriptome data under sulfur deficiency in *O. sativa* and *S. viridis*

To obtain a global view of the transcriptome responses to sulfur deficiency in the model C_3_ and C_4_ monocot plant species, we performed RNA sequencing (RNA-seq) of 27 days old *O. sativa* (C_3_) and *S. viridis* (C_4_) grown for 20 days under complete nutrient solution or S-deficiency (Fig. 1A). Three biological replicates were collected separately for shoots and roots, where three plants were pooled together. The RNA was processed and sequenced using the Illumina sequencing platform at Novogene, UK. At least 30 million high-quality reads per sample were generated and mapped to the *Oryza sativa kitaake* (Phytozome V3.1) or *Setaria viridis* (Phytozome V2.1) reference genomes [21] using Hisat2 [22]. Over 95% of the reads were uniquely mapped and only the uniquely mapped reads were further counted at the gene level with featureCounts [23].

To identify differentially expressed genes (DEGs) we used DESeq2 [24] and limma [25] (see Methods). Then, we applied intersection analysis of these results to identify a stringent list of differentially regulated genes under S-deficiency in both species. This analysis revealed in total 236 DEGs in *O. sativa* (Supplemental Table 1), and 565 DEGs in *S. viridis* (Supplemental Table 2) to be regulated under S-deficiency in both roots and shoots (Fig. 1C, Supplemental Tables 1 and 2). However, for further analysis we consider only genes that had orthologous genes in *Arabidopsis thaliana* to enable a direct comparison among the species.

As a control, we compared the RNA-seq results by qPCR using RNA from an independent experiment. We used 8 genes for *O. sativa* and 16 genes for *S. viridis*, and most of them had high R^2^ confirming the RNA-seq results (Supplemental Fig. 2A and B).

### SLIM1/EIL3 is differentially expressed in both *O. sativa* and *S. viridis*

**Fig. 2:**
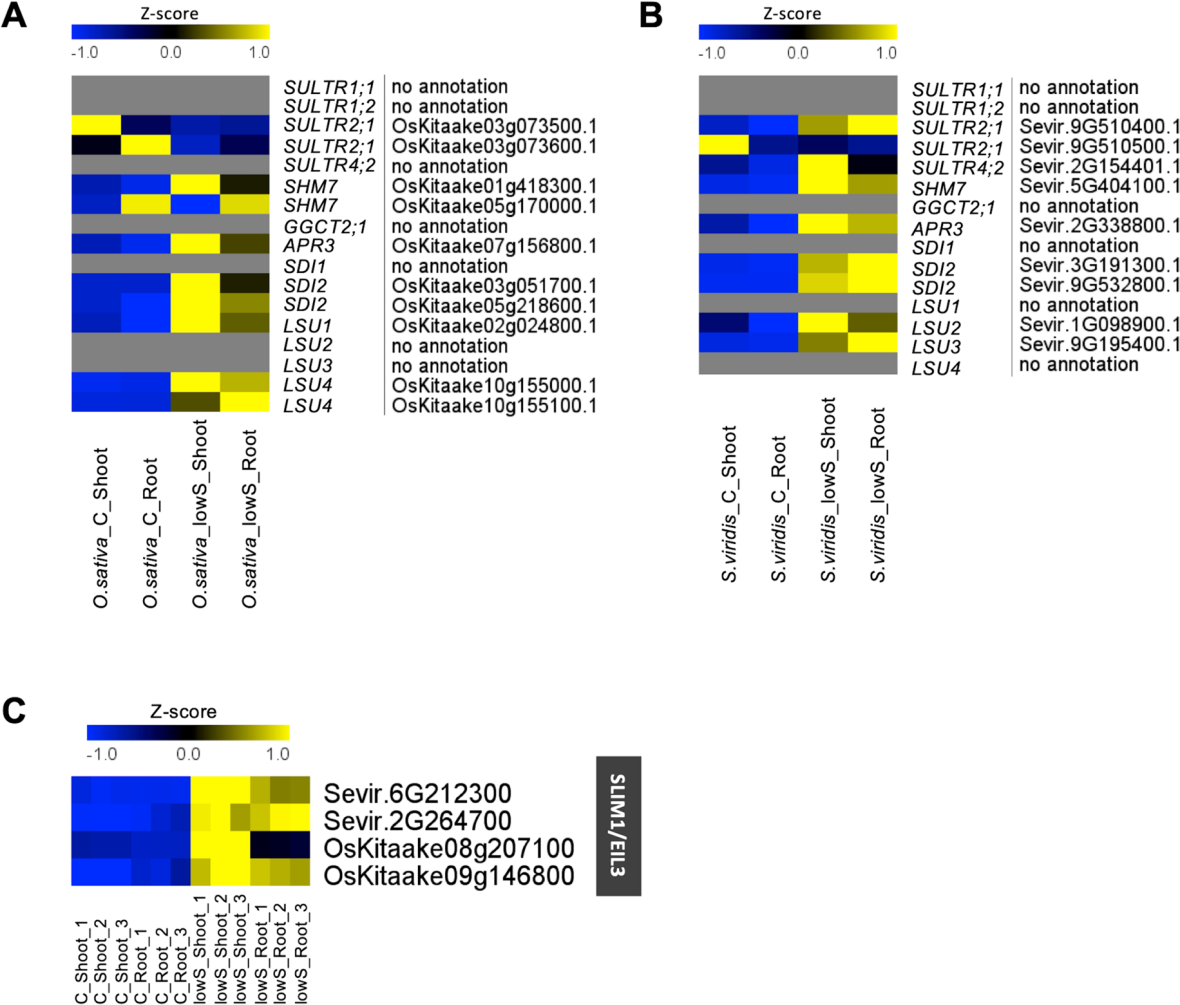
Regulation of Arabidopsis S-deficiency marker genes in *O. sativa* and *S. viridis*. **A** Heatmap of mean z-scores of sulfate transporters, OAS cluster genes, and LSU-genes in *O. sativa*. **B** Heatmap of mean z-scores of sulfate transporters, OAS cluster genes, and LSU-genes in *S. viridis*. **C** Heatmap of all replicates for the *SLIM1/EIL3* gene in *O. sativa* and *S. viridis*. Full media corresponds to 1000 µM SO_4_^2−^ (**C**), and S deficiency corresponds to 12.5 µM SO_4_^2−^ (lowS). Annotation according to *Phytozome* (*OsativaKitaake_499_v3*.*1* and *Sviridis_500 v2.1*)

To validate the quality of the gene expression profiles we obtained, we specifically examined the expression patterns of genes for which transcript levels were previously reported to be differentially regulated during S starvation in *Arabidopsis* (Supplemental Table 3) [10, 11]. However, we noticed that some key genes known to be upregulated under S-deficiency in Arabidopsis (e.g. LSU genes) were not present in our results. Similarly, OAS cluster genes, known to correlate with increased O-acetylserine (OAS) levels, which are also increased during S-starvation [26], were not all present in the DEGs in both species (Supplemental Table 3). Thus, we searched for these genes specifically in the annotations and found that many of them did not have ortholog gene match for *Arabidopsis thaliana* (Fig. 2A and B). For instance, *SULTR1;1* and *SULTR1;2*, the two main sulfate uptake transporters [27], are not present in the *Phytozome* annotation (OsativaKitaake_499_v3.1). However, when we search for orthologs for *A. thaliana SULTR1;2* [28], we found that Os03g0195800 (LOC4331935) from *Oryza sativa japonica* had 74% sequence identity, and this gene corresponded to LOC_Os03g09970 or OsKitaake03g073800, and was annotated as *SULTR1;3* (AT1G22150) in the *Phytozome* annotation. This gene (OsKitaake03g073800) is significantly 4-fold upregulated under S-deficiency (Supplemental Table 1), but *SULTR1;3* hasn’t been reported to be significantly upregulated in *A. thaliana* under S-deficiency [10, 11]. Likewise, *SDI1* gene is not annotated, but there are two *O. sativa Kitaake* genes that are annotated as *SDI2* (OsKitaake05g218600 and OsKitaake03g051700) and both are 3 to 12-fold upregulated under S-deficiency (Supplemental Table 1). We found similar situation in *S. viridis*. The most significantly upregulated gene under S-deficiency was Sevir.9G510300.1 (Supplemental Table 2) which in current *Phytozome* annotation (Sviridis_500 v2.1) is annotated as *SULTR1;3*, but in the OrthoDB v11 [28] is annotated as *SULTR1;2*-like (LOC117839334). Therefore, absence of some key S-deficiency genes from our results, might be an issue of incorrect annotation. However, to simplify the interpretation, we considered only genes for which we found annotations in the current *Phytozome* version for *O. sativa Kitaake* (499 v3.1).

Next, we checked the expression of *SLIM1/EIL3*, encoding the main transcription factor known to regulate S starvation response genes in *Arabidopsis*, while not being transcriptionally regulated under S-deficiency itself in *A. thaliana* [10, 11]. Interestingly, *SLIM1/EIL3* was differentially regulated in both species (Fig. 2C). The transcripts, *OsKitaake08g207100.1* and *OsKitaake09g146800* for *O. sativa*; and *Sevir.6G212300.1* and *Sevir.2G264700.1* for *S. viridis* mapped to the *SLIM/EIL3* gene. In *S. viridis* both transcripts were highly up-regulated in both roots and shoots, while in *O. sativa* the *OsKitaake08g207100.1* transcript was highly induced only in the shoots. These findings suggest that although SLIM1/EIL3 is the central TF regulating S starvation response genes, it is differently regulated in diverse species and tissues and probably also has different mechanisms of action.

### Only 58 genes are commonly regulated by S-deficiency in *O. sativa* and *S. viridis*

**Fig. 3:**
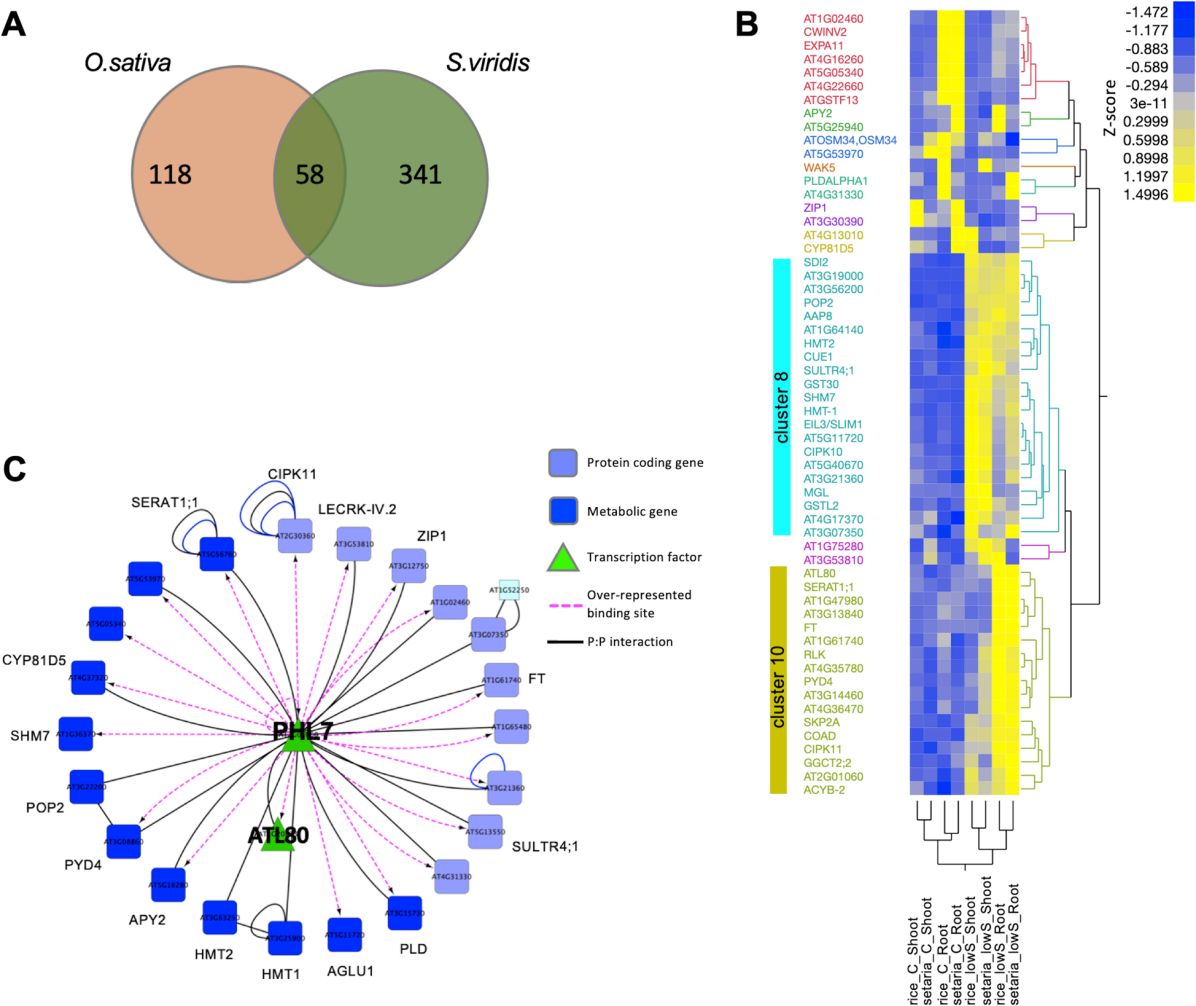
Intersect between DEGs regulated under S deficiency in *O. sativa* and *S. viridis*. **A** Venn diagram of DEGs between *O. sativa* and *S. viridis* under S deficiency. **B** Hierarchical clustering of 58 intersecting DEGs between *O. sativa* and *S. viridis* plants. Different colors of the genes correspond to different clusters (1–10). **C** Multinetwork of the 58 intersect genes created by the Gene Networks analysis tool in VirtualPlant software (http://virtualplant.bio.nyu.edu/cgi-bin/vpweb/; Katari et al., [29]), centered at myb-like transcription factor PHL7 (AT2G01060). Plants were grown hydroponically on full media (**C**) and S-deficient media (lowS). For the transcriptomics (RNA-Seq) shoots and roots were harvested separately, 3 biological replicates for each. DEGs are defined as transcripts that were significantly regulated under S-deficiency (lowS), when intersecting results from two different approaches, DESeq2 and limma analysis (adjpval < 0.01 see Methods). Hierarchical clustering (HC) average was performed on z-scores of DEGs

One of the aims of this study was to identify common molecular signatures that regulate plant responses to S-deficiency. Thus, we intersected all DEGs for *O. sativa* and *S. viridis* mapped to the AT IDs, and found that only 58 genes overlap (Fig. 3A). Among the 58 overlapping genes we found several genes, known previously to be differentially regulated in *Arabidopsis thaliana* under S starvation, including *SHM7*, *SDI2*, *SERAT1;1*, and *SULTR4;1* (Fig. 3B). To determine possible connections of the intersect genes with other genes, we used Gene Network analysis performed in VirtualPlant platform that uses multiple sources of information on gene, protein, and RNA interactions [29]. The generated network contains 25 gene nodes that are connected to each other by 50 edges, representing regulatory relationships, such as predicted over-representation of transcription factor binding sites within the promoter region of the target gene (Fig. 3C). Among the 26 genes, two transcription factors (TFs). PHR1-LIKE 7 (PHL7, AT2G01060) and ATL80 (AT1G20823). were represented, with PHL7 taking the central place in the network, as most of the genes had over-represented binding site for AT2G01060 in their promoters (Fig. 3C). Both TFs were not previously described to be associated with S-starvation response in *A. thaliana*.

### GO categories and metabolic pathways in *O. sativa* and *S. viridis* display little overlap under S-deficiency

**Fig. 4:**
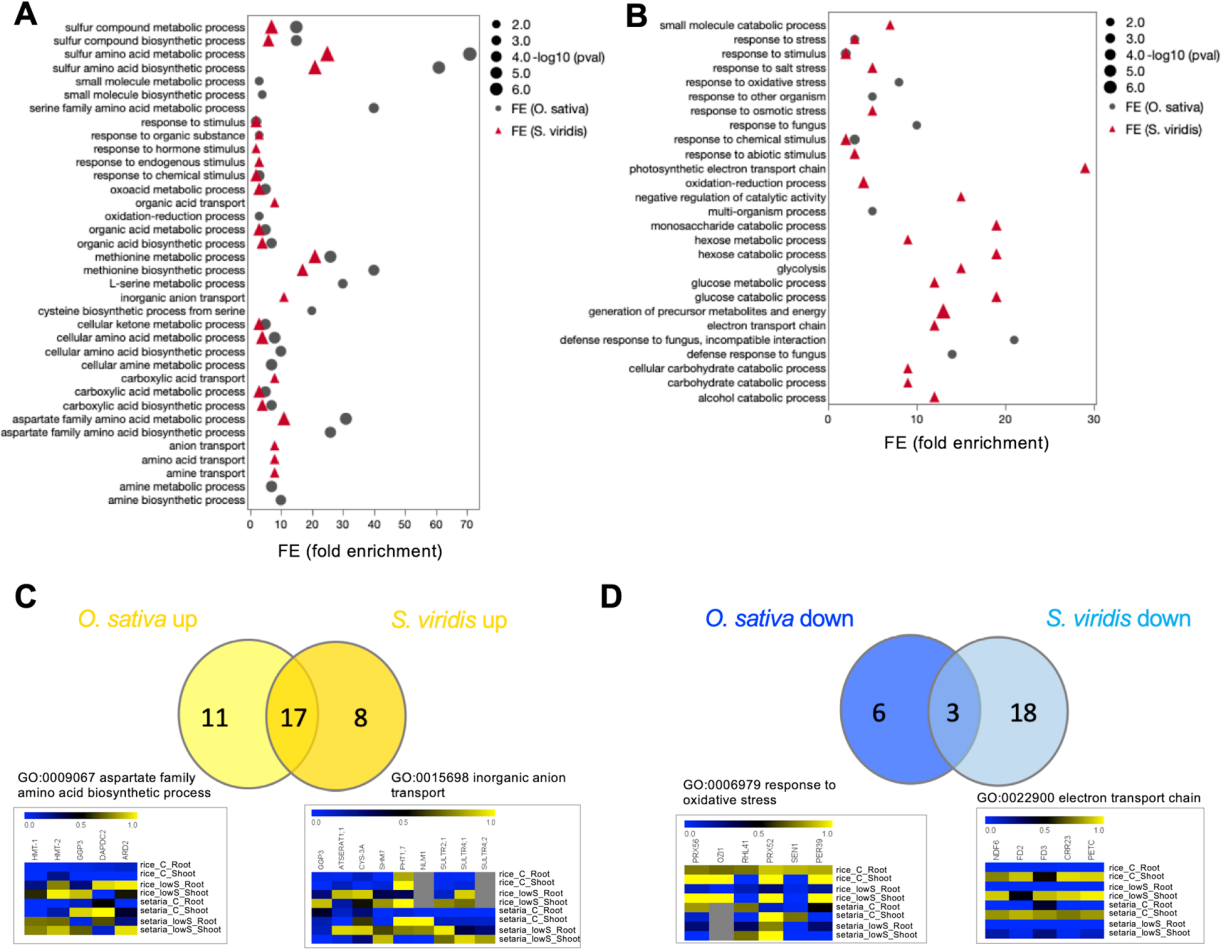
GO enriched terms in *O. sativa* and *S. viridis* under S deficiency. **A** Functional gene ontology (GO) enrichment analysis of upregulated DEGs in *O.sativa* and *S. viridis*. **B** Functional gene ontology (GO) enrichment analysis of downregulated DEGs in *O.sativa* and *S. viridis*. **C** Intersecting GO terms for the up-regulated DEGs in *O.sativa* and *S. viridis*. **D** Intersecting GO terms for the down-regulated DEGs in *O.sativa* and *S. viridis*. Functional gene ontology (GO) enrichment analysis was performed with Biomaps app [29] in VirtualPlant (http://virtualplant.bio.nyu.edu/)

**Fig. 5:**
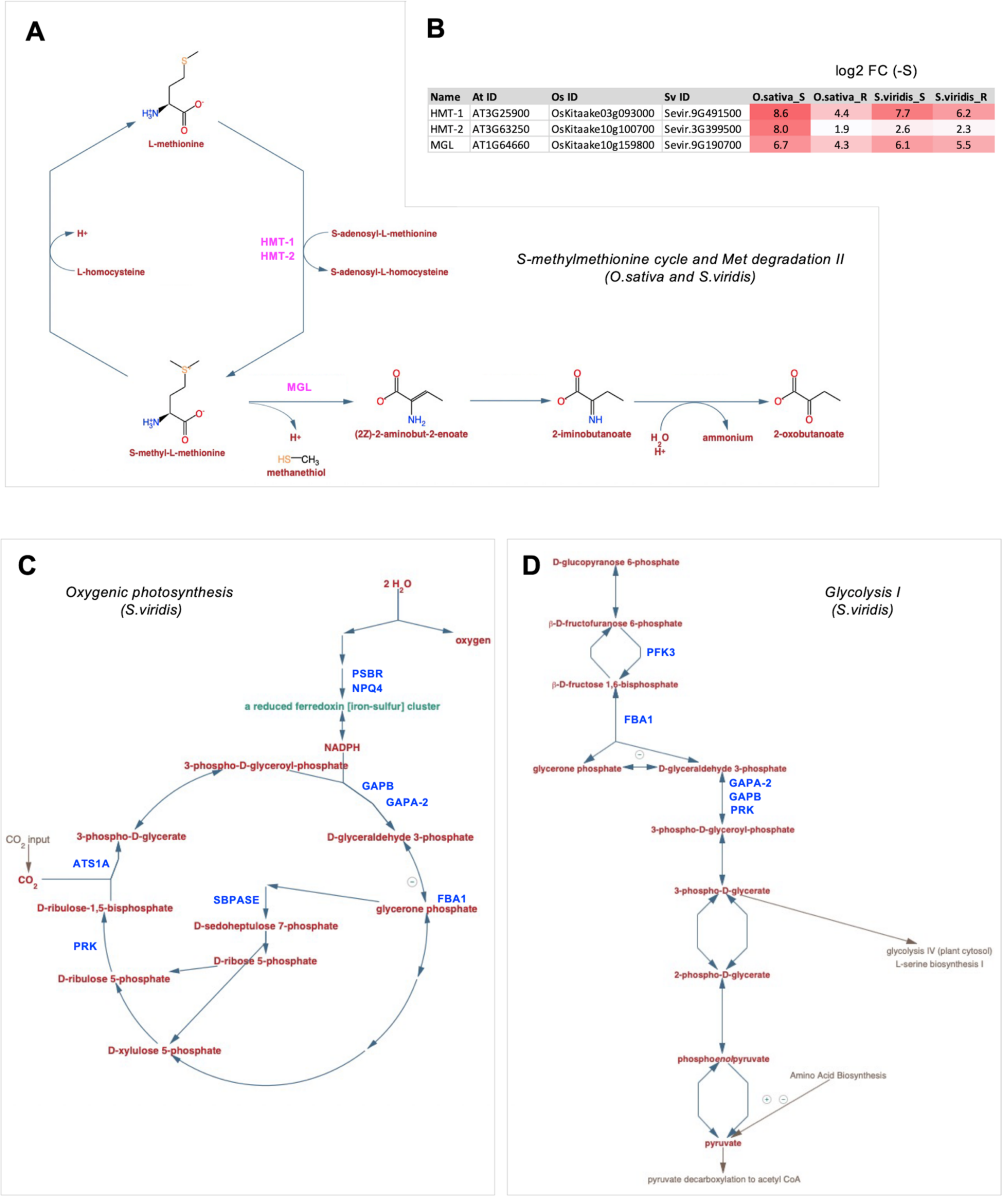
Pathway analysis of DEGs under S-deficiency, in *O. sativa* and *S. viridis*. **A*** S-methylmethionine cycle* and *Met degradation II* were the most significantly altered pathways in the up-regulated DEGs in both species (complete list in Supplemental Table 4). Up-regulated genes are shown in magenta. **B** Regulation by S-deficiency of *HMT-1* and *HMT-2* genes from the S-methylmethionine cycle pathway and *MGL* from Met degradation II in *O. sativa* and *S. viridis*, (log2 fold change) separately in shoots (S), and roots (R). **C** The pathway *oxygenic photosynthesis* was significantly down-regulated specifically in *S. viridis*, where 8 genes were down-regulated (in blue). **D** The pathway *glycolysis II* was significantly altered only in *S. viridis*, where 5 genes were down-regulated (in blue). Pathways were downloaded from https://plantcyc.org

We next examined the functions of genes enriched within the identified DEGs using Gene Ontology (GO) (see Methods), using the Arabidopsis IDs. In the up-regulated gene list from *O. sativa* we identified 28 significantly enriched functional categories, while 25 categories were found in *S. viridis* (Fig. 4A, Supplemental Tables 4 and 5). Fewer categories, 9 and 21, for *O. sativa* and *S. viridis*, respectively, were enriched among the down-regulated DEGs (Fig. 4B, Supplemental Tables 6 and 7). The significantly enriched functional categories differed between the two groups. Namely, under S-deficiency 17 significantly enriched functional categories overlapped among the up-regulated genes in *O. sativa* and *S. viridis*, while 11 were specific for *O. sativa*, and 8 categories were specific to *S. viridis* (Fig. 4C). For instance, the category “aspartate family amino acid metabolic process” was enriched in *O. sativa*, but not in *S. viridis*. On the other hand, “inorganic anion transport” was enriched in *S. viridis*, but not in *O. sativa* plants. Interestingly, one of the 17 shared enriched GO categories (Fig. 4C) was “methionine metabolic process”. This category has 3 genes, including Homocysteine S-methyltransferase 1 (*HMT1*), *HMT2*, and methionine gamma-lyase (*MGL*), involved in synthesis of methionine from homocysteine as well as its degradation to methanethiol (Fig. 5A and B).

More pronounced differences were identified for the down-regulated genes, where only 3 categories overlapped and 6 were *O. sativa* specific, while 18 were *S. viridis* specific (Fig. 4D). Here, “response to oxidative stress” was enriched only in *O. sativa*, while “electron transport chain” only in *S. viridis*. These results indicate that although many functional categories are overlapping in response to S-starvation, a large number of GO categories are specific for given species, suggesting that *O. sativa* and *S. viridis*, or possibly generally C_3_ and C_4_ species, have distinct molecular responses to S-deficiency.

Next, we examined the metabolic pathways that were significantly regulated under S-deficiency in *O. sativa* and *S. viridis* plants (Supplemental Fig. 3). In *O. sativa*, using the upregulated DEGs we identified 12 pathways significantly altered, while 11 were specific for *S. viridis* and 6 pathways were shared (Supplemental Fig. 4A). The shared pathways included S-methylmethionine cycle, methionine biosynthesis II, and methionine degradation II, indicating that methionine metabolism might be crucial for both monocots to cope with S deficiency. Indeed, the three genes involved in Met metabolism in both species and both organs were up-regulated from 1.9 to 8.6 (log2FC) depending on the organ (Fig. 5A and B). Downregulated genes could be assigned to many different pathways. Namely, 7 pathways were significantly downregulated in *O. sativa*, but 15 in *S. viridis* (Supplemental Fig. 4B). Interestingly, only one pathway, the “4-hydroxyphenylpyruvate biosynthesis” was shared between *O. sativa* and *S. viridis* (Supplemental Fig. 4B). Similarly to the overrepresented GO functional categories, the findings in the two model species indicate that many metabolic pathways might be differentially altered under S-deficiency in C_3_ and C_4_ species. For instance, “oxygenic photosynthesis” and “Glycolysis I” are specifically downregulated only in C_4_* S. viridis*, where 8 and 5 genes were down-regulated, respectively (Fig. 5C and D, Supplemental Fig. 4B).

### Specific response to S-deficiency in shoots and roots of *O. sativa* and *S. viridis*

Previous studies in Arabidopsis showed that the response to S deficiency differs in shoots and roots [10]. To assess whether also in the monocot species the transcriptomic networks and pathways differentially altered under S-deficiency diverge in shoots and roots, we analyzed the data separately (see Methods). In rice shoots we identified 291 upregulated DEGs and 10 GO enriched biological terms, while for rice roots we identified only 115 upregulated DEGs and 35 GO terms (Supplemental Fig. 5). Only one GO term was specific for shoots ("response to stress”), 9 terms were shared, and 26 GO terms were specific for roots.

In *S. viridis*, the number of up-regulated DEGs was much larger in the shoot than in the root, 900 vs.119, respectively. Similarly, for shoots we obtained 44 enriched GO terms, while only 8 terms were found to be enriched in roots (Supplemental Fig. 6).

Next, we compared the enriched GO terms between the two monocot species. We intersected the 10 GO terms for up-regulated DEGs in *O. sativa* shoot with the 44 GO terms for up-regulated DEGs in *S. viridis* shoot, and found only 2 GO terms that overlapped, which were “response to stress” and “response to stimulus” (Supplemental Fig. 7A). For the GO terms up-regulated in root DEGs, 6 common GO terms were identified (Supplemental Fig. 7B), while 7 GO terms intersected for the down-regulated GO terms in the shoot of *O. sativa* and *S. viridis* (Supplemental Fig. 7C). These findings thus reveal that the two monocot species have specific above- and below-ground transcriptional reprogramming in response to S-deficiency and that the responses differ also in the individual species.

### Metabolite analysis

**Fig. 6:**
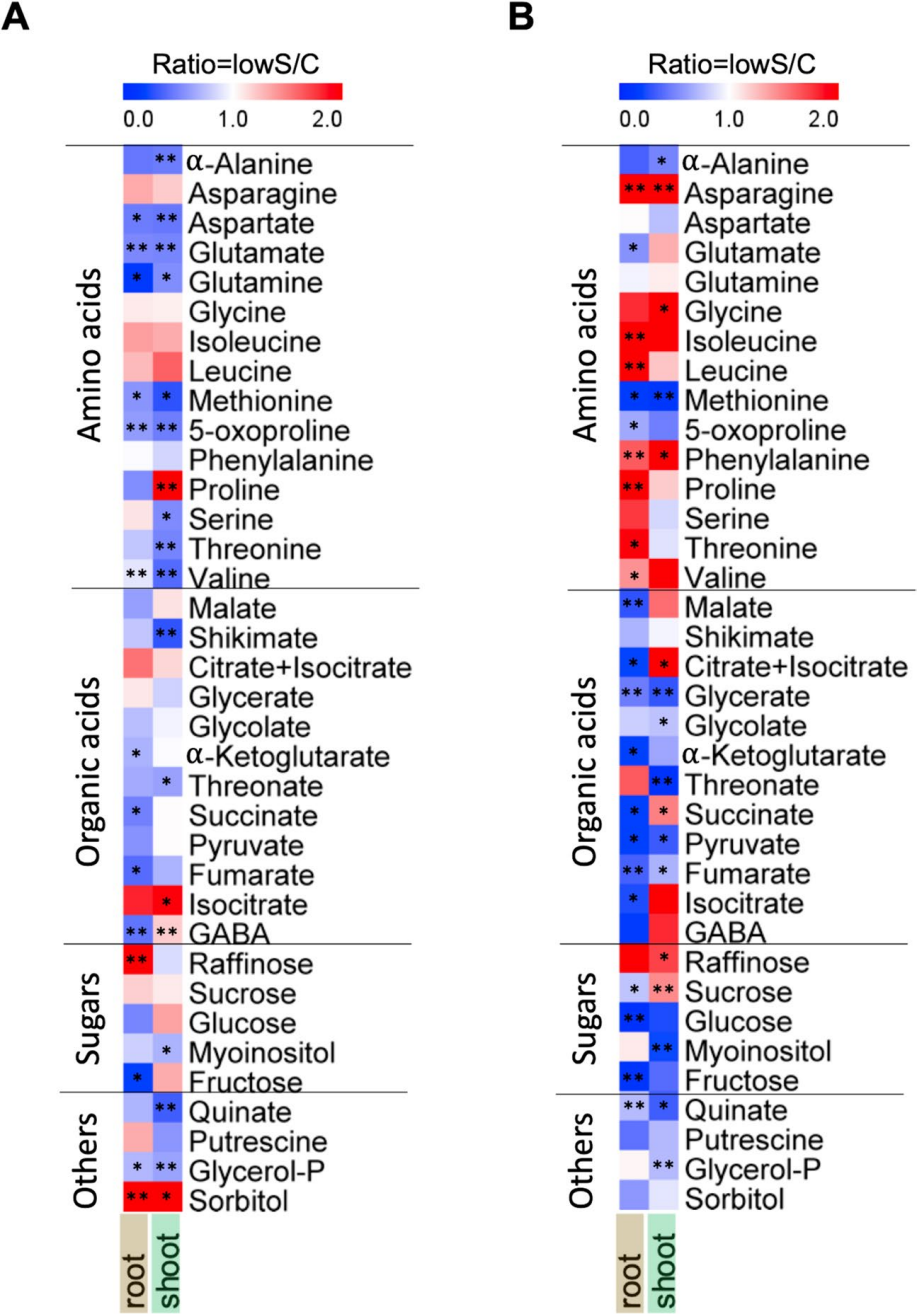
Metabolite profiling in *O. sativa* and *S. viridis* under S-deficiency. Heatmap of the changes in metabolite accumulation in roots and shoots under S-deficiency compared to control conditions in *O. sativa* (**A**) and *S. viridis* (**B**). Mean values from 3 biological replicates were used to compute the ratio between the two conditions (lowS/C). The blue color represents the trend of decrease, while the red color represents an increasing trend. Asterisks indicate statistically significant differences, according to anova statistics with *P* < 0.05 (*n* = 3)

S-deficiency causes not only transcriptional reprogramming, it has also a significant effect on plant metabolome. To identify similarities and differences in the metabolite profile of *O. sativa* and *S. viridis* under S-deficiency, we used the same tissue as collected for transcriptomic analysis and quantified 36 primary metabolites (Fig. 6). Among the amino acids, an obvious difference was the increased accumulation of branched-chain amino acids (BCAAs; leucine, isoleucine, and valine) in *S. viridis* roots under S-deficiency, compared to *O. sativa* (Fig. 6A and B). Further significantly accumulated amino acids in *S. viridis* roots were proline and threonine, whereas phenylalanine and asparagine were increased in both roots and shoots (Fig. 6). On the other hand, in *O. sativa* most significant changes were a lower accumulation of several amino acids, such as aspartate, glutamine, glutamate, methionine, 5-oxoproline, and valine in both tissues; and α-alanine, serine, and threonine only in shoots (Fig. 6A), which contradicts the usual accumulation of amino acids measured in a number of different plant species. The only significantly more highly accumulated amino acid in *O. sativa* was proline in the shoots (Fig. 6A). Organic acids were mostly significantly less accumulated by S-deficiency in both species, with exception of GABA that was lower in roots and higher in shoots of *O. sativa* (Fig. 6A). The sugars showed a similar trend, but more significant changes were observed in *S. viridis*, where glucose and fructose were less abundant in the roots, while myoinositol accumulated more highly in the shoots (Fig. 6). Sucrose showed an opposite trend in roots and shoots of *S. viridis*. Finally, in the last subset of metabolites, we observed a significant accumulation of sorbitol in both tissues of *O. sativa* (Fig. 6).

## Discussion

### Sulfur compounds under S deficiency in *O. sativa* and *S. viridis*

S deficiency has significant inhibitory effect on total biomass accumulation in both *O. sativa* and *S. viridis* (Supplemental Fig. 1A). Similar observations were reported in multiple plant species, including *A. thaliana* [30], *O. sativa* Zhonghua 11 [31], maize [32], tomato [33], and barley [34]. Organic and inorganic sulfur pools underwent sharp decrease in both roots and shoots, slightly more pronounced in the roots (Supplemental Fig. 1B). Sulfate, a common inorganic sulfur storage pool in plant cells, decreased 65–83% depending on the tissue and species (Supplemental Fig. 1C). This considerable decrease was previously reported for *A. thaliana* [10], tomato [33], and maize [32]. Interestingly, cysteine, which represents the first organic sulfur compound of primary sulfur assimilation pathway, was not altered by S deficiency in *O. sativa*, but it was strongly reduced in both shoots and roots of *S. viridis* (Supplemental Fig. 1D). In *A. thaliana* shoots, slight but significant reduction of about 20% was previously reported for cysteine [10]. These differences in cysteine content under S deficiency in different plants, suggest that different regulatory mechanisms controlling cysteine homeostasis operate in distinct species. Glutathione on the other hand, was reduced in both species and both organs, but in *S. viridis* this reduction was again massive, by about 95% (Supplemental Fig. 1E). Likewise, in *A. thaliana*, GSH content shows substantial reduction in S starved seedlings [12], and S starved shoots [10]. The tripeptide glutathione (GSH) is the most abundant low molecular weight thiol in plants, and plays crucial role in the defense against various biotic and abiotic stress conditions, as well as maintaining redox homeostasis [35]. In C_3_ plants, including *A. thaliana*, GSH triggers the demand-driven control of sulfate assimilation [35, 36]. However, in maize which represents C_4_ plant, it was observed that cysteine is the major regulator of sulfate assimilation [37]. Recently, we showed that GSH seems to be the signal for demand-driven regulation of sulfate assimilation in both *O. sativa* and *S. viridis* and that these species differ in the response to cysteine and GSH feeding [20]. Thus, cysteine and GSH metabolism in the two monocot species is differently regulated in reaction to a number of different triggers. The difference between *O. sativa* and *S. viridis* might be at least partly triggered by the different photosynthetic mechanism, in analogy to the dicot C_4_ species, which have higher concentration of reduced S compounds [16, 17] and thus might be more sensitive to S deficiency, as is *S. viridis* in comparison to *O. sativa*.

### Transcriptome response to S deficiency is largely species specific

Using a stringent approach, the intersect of two methods (DESeq2 and limma), we identified 236 and 565 DEGs under S deficiency in *O. sativa and S. viridis*, respectively (Fig. 1C, Supplemental Tables 1 and 2). However, only 58 DEGs were common between *O. sativa* and *S. viridis* (Fig. 3), showing that relatively high number of DEGs are specific for the individual species. Among the 58 genes that represent the core S-deficiency response in the monocots, particularly interesting were those found in clusters 8 and 10 of the clustering analysis, i.e., genes upregulated in both species and both tissues under S-deficiency, and up in both species only in roots, respectively (Fig. 3B). Here, several genes known to be up-regulated under S-deficiency in *A. thaliana* [10, 12, 13], were present, including *SDI2*, which together with *SDI1* are involved in partitioning sulfur among metabolite pools during S- deficiency, controlling the glucosinolate biosynthesis in *Arabidopsis* under low S conditions [38, 39]. Similarly, *SHM7/MSA1,* which regulates S-adenosylmethionine (SAM) production and DNA methylation including the methylation of S-deficiency responsive genes, such as the main sulfate transporters *SULTR1;1* and *SULTR1;2* [40], was included in cluster 8 (Fig. 3B). Other common genes present in cluster 8 are the previously discussed *HMT1*, *HMT2*, and *MGL*, as well as *SLIM1/EIL3*. Several other unknown genes were also present in the cluster 8 and 10, which were previously identified to be up-regulated under S-deficiency in Arabidopsis or other species [10, 11, 13], but their significance in S-deficiency response is not known.

When we compared the 58 genes with genes known to be regulated by S deficiency in *A. thaliana* [10, 12], we found that many genes did not have ortholog gene match for *A. thaliana* according to the current phytozome resources (Fig. 2A and B). However, we found that some genes, such as *SULTR1;2* and *SDI1* in fact are annotated to other transporters or redundant genes. Taking that into account, most of the marker genes of sulfate deficiency described for Arabidopsis are differentially regulated also in *O. sativa* and *S. viridis.*

In *O. sativa*, another transcriptional study was recently reported, which identified over 1500 DEGs in shoots, and over 6500 DEGs in the roots [41]. Most of the DEGs identified in our study of *O. sativa kitaake* have been regulated by S deficiency in the experiments by [41] as well. However, our analysis identified much fewer DEGs, which is probably due to the higher stringency and threshold used in our study.

### GO categories and metabolic pathways in *O. sativa* and *S. viridis* under S-deficiency

Low conservation of the response to S-deficiency between the two monocot species was further corroborated by comparison of the enriched GO categories. Namely, 28 biological GO terms for the up-regulated DEGs were obtained specifically in *O. sativa* and 25 in *S. viridis*, but only 17 GO terms overlapped between the two species (Fig. 4). As expected, among the conserved functions, terms involving S metabolism and amino acid metabolism were the most significant. This is reflected also in the metabolic pathway of conversion of homocysteine to methionine and its degradation to methanethiol being induced in both species (Figs. 4A and 5A). Among the *O. sativa* specific GO categories, terms connected to amino acid metabolism were prevailing (Fig. 4A). When comparing our findings with previously reported transcriptomic analysis in a different rice cultivar Zhonghua 11 [41], we found only a minor overlap of GO terms, which however, included Met metabolism. It has to be noted, though, that the rice variety and growing conditions in both studies are different. In *S. viridis* most of the specific categories involved the term “transport” (Fig. 4A and B). C_4_ photosynthesis requires massive traffic of metabolites between the MCs and BSCs [42]. Additionally, S assimilation and Cys production is also exclusively localized in BSCs of C_4_ monocots [15], which may involve further traffic of metabolites under S-deficiency. However also in rice sulfate assimilation is preferentially expressed in BSCs [43], therefore, the impact of localization of sulfate assimilation on transcriptional networks still needs to be elucidated. Comparison of the GO terms among the down-regulated DEGs identified 9 GO categories for *O. sativa* and 21 for *S. viridis*, with only 3 terms overlapping (Fig. 4D). The specific terms for *O. sativa* suggested that S-deficiency in rice decreases the defense response to other organisms, as recently suggested [44]. The enriched GO terms in *S. viridis* imply that S-deficiency in C_4_ plants has stronger impact on down-regulation of the photosynthesis, possibly because of a higher foliar content of GSH at control conditions and a greater impact of S deficiency on its concentration (Supplemental Fig. S1). Although, S-deficiency in rice significantly reduces chlorophyll content and photosynthetic efficiency [45], we found enriched GO terms related to photosynthesis only in *S. viridis* (Fig. 4).

### Transcription factors in response to S-deficiency

Interestingly, SLIM1/EIL3, the central transcriptional factor of S deficiency, which is not transcriptionally regulated in *A. thaliana* [10, 11], seems to be present in two isoforms in both *O. sativa and S. viridis*, and both are significantly upregulated under S deficiency (Fig. 2C). Similarly, in tomato, *SLIM1/EIL3* (*Solyc01g006650*) was significantly upregulated in both shoots and roots under S starvation [33], which together with our findings suggest that the key TF regulating multiple S starvation response genes is differently regulated in diverse species and tissues and probably also has different mechanisms of action. Nevertheless, both SLIM1 isoforms from *O. sativa* were able to complement the loss of Arabidopsis SLIM1 [11], so that some conservation of function must exist.

Our network analysis pin-pointed additional two TFs that haven’t been previously associated with S-starvation response in *A. thaliana*. The function of PHL7 is unclear, except that due to its sequence similarity to PHR1 it might be involved in phosphate (Pi) long-term starvation response, but it is not transcriptionally regulated by Pi starvation [46]. ATL80 is a RING E3 ubiquitin ligase involved in response to cold and regulation of flower development and phosphate transport [47]. Both genes haven’t been previously associated with S-starvation response, and are potentially novel components of S-deficiency response to modulate plant signaling and development. In our analysis both genes were significantly upregulated by S starvation in roots, and PHL7 in both roots and shoots of both species (cluster 10 of Fig. 3B). Furthermore, both genes are associated with Pi homeostasis, and could be new regulators, besides PHR1 [48], that modulate and coordinate the two major nutrient homeostasis and starvation responses. Whether these factors are specific to monocots or have function also in Arabidopsis needs to be explored, especially as they have not been among the genes regulated by S deficiency in previous reports [10].

### Metabolic signatures under S-deficiency in *O. sativa* and *S. viridis*

Metabolite analysis revealed further major differences in response to S-deficiency between the two monocot species. A striking difference in the accumulation of amino acids between the two species was the accumulation of the BCAAs; leucine, isoleucine, and valine, only in *S. viridis* (Fig. 6). Osmotic-stress-induced accumulation of BCAAs is dependent on ABA-regulated protein degradation in *A. thaliana*, unlike the proline accumulation that is due to de novo synthesis [49]. In rice, DROUGHT-INDUCED BRANCHED-CHAIN AMINO ACID AMINOTRANSFERASE (OsDIAT) mediates the accumulation of BCAAs in response to drought stress; and rice plants overexpressing OsDIAT have increased accumulation of BCAAs and are more tolerant to drought stress [50]. Significant accumulation of BCAAs only in *S. viridis* suggests that C_4_ monocots might have more efficient mechanism to cope with osmotic-stress occurring due to sulfate starvation. In contrast to *A. thaliana* and other species, in *O. sativa* most amino acids were significantly reduced and not increased (Fig. 6A). A typical feature of S-limitation in different plant species is poor utilization of N, because of the down-regulation of nitrate reductase (NR) and glutamine synthetase activity that leads to accumulation of nitrate and related amino acids [51, 52]. In *O. sativa*, most amino acids were reduced, including glutamine and glutamate that are possibly linked to the known effect that S-deficient plants are unable to properly utilize N and subsequently become N deficient [53].

Most of the organic acids were less accumulated in both shoots and roots in both species, but the effect was more significantly pronounced in *S. viridis* roots. These included metabolites related to TCA cycle, such as pyruvate, fumarate, and glycerate, that were significantly down-regulated only in the C_4_ plant (Fig. 6). Pyruvate, which is the final metabolite of glycolysis, a pathway that was significantly down-regulated in our transcriptome analysis (Fig. 4B), was less abundant in both roots and shoots in S-deficient *S. viridis* plants (Fig. 6B). However, amino acids derived from pyruvate were differently accumulated, while α-alanine was less abundant in shoot, leucine and valine accumulated more in the roots. These findings suggest different demand for pyruvate for amino acids synthesis and TCA cycle in the roots and shoots of C_4_ plants. Similar finding was observed in another C_4_ halophyte species under salt stress [54], suggesting that S-deficiency and salt stress might have similar cellular osmotic alteration and similarly affect TCA metabolites in C_4_ plants.

### Integration of transcripts and metabolites under S deficiency in *O. sativa* and *S. viridis*

Correlation of transcripts and metabolites resulted in distinct networks in *O. sativa* vs *S. viridis* (Supplemental Fig. 8). For instance, in *O. sativa*, the most significantly enriched GO term in the larger network was “response to stress” (Supplemental Table 8). S-related GO terms were found for the smaller network connecting 4 amino acids (glutamine, aspartate, α-alanine, and 5-oxoproline), sorbitol and 28 genes, of which two encode TFs (Supplemental Fig. 8A, right). In *S. viridis*, similarly, several sub-networks were obtained, that had also large number of enriched GO categories, related to transport and photosynthesis (Supplemental Table 9). S-related GO terms were found for the largest network that included 7 amino acids (leucine, isoleucine, serine, proline, asparagine, phenylalanine, and glycine), sorbitol, and 263 genes (Supplemental Fig. 8B, left). Since Met metabolism pathway is affected in both species (Fig. 4A), we compared the sub-networks of genes directly connected to Met. Interestingly, in *O. sativa*, Met was directly connected by negative correlation with isocitrate and 5 genes (*LSU1, APR3, SIP1, GSTL2*, and *AT5G35320*), while in *S. viridis* it was connected with α-alanine and 7 genes (*CYP81D5, ALAAT2, GNAT6, NHL13, XTH5, GSTF13*, and *AT1G01240*) were positively correlated with Met (Supplemental Fig. 8A and B). These findings again suggest that network modules operate differently in *O. sativa* vs *S. viridis* under S-deficiency. How far this difference is governed by the different photosynthetic mechanisms, C_3_ and C_4_, respectively, need to be further investigated.

## Conclusions

In conclusion, we demonstrate that the transcriptional response to S deficiency in two model monocot species shows distinct patterns that are probably linked to different photosynthetic type of C_3_ and C_4_, which has different consequences on plant metabolism and coping with S deficiency. Our comprehensive transcriptomic analysis in roots and shoots of *O. sativa* and *S. viridis* under S deficiency revealed common transcriptional upregulation of key enzymes involved in Met degradation, which is crucial for recycling of reduced S into the primary metabolism. Furthermore, our finding of distinct GO terms under S starvation, suggest that S-deficiency probably has different physiological consequences and nutrient homeostasis might be under diverse control mechanisms. In conclusion, our analysis will help understanding S starvation response in other crops and model plant species beyond Arabidopsis, and can contribute to future designing of crops that cope better with nutrient deficiency environment.

## Methods

### Plant material and growth conditions

The C_3_ monocot species *Oryza sativa* subsp. *japonica* cv. Kitaake (further denoted *O. sativa*), obtained from Dr. Pamela Ronald (University of California, Davis, USA). and the C_4_ monocot *Setaria viridis* reference line ‘A10.1’ (*S. viridis*), obtained from Dr. Thomas Brutnell (Donald Danforth Plant Science Center, St. Louis, USA), were used as the model system. For germination, *S. viridis* seeds were pre-treated with a 5% aqueous solution of hickory liquid smoke (Wright's® Liquid Smoke, B&G Foods, Parsippany, New Jersey, USA) for 24 h at 31 ºC, followed by thorough rinsing with ultra-pure water, to overcome dormancy [55]. Surface disinfestation of seeds from both species was performed by incubation in freshly prepared 0.6% sodium hypochlorite (NaOCl) solution for 20 min at 21 ºC, followed by thorough rinsing with ultra-pure water. Germination took place in polystyrene Petri dishes lined with three layers of Whatman’sⓇ filter paper saturated with ultra-pure water. The containers with the Petri dishes were accommodated in a controlled environment set to long-day photoperiod (16 h light/8 h dark), with a temperature of 31ºC light/21ºC dark, and a photon flux density of approximately 140 μmol m^−2^ s^−1^ at the leaf blade level provided by 16 dimmable fluorescent bulbs (Philips F32T8/TL741/32W). Seven days after imbibition, seedlings with the first expanded leaf blade, i.e., at the developmental stage 1.10 in *S. viridis* [56], and stage 1.11 in *O. sativa* [57], were transferred to a static hydroponic system to grow for 20 days under the same conditions. Mineral nutrition was provided using a modified half-strength Hoagland solution. The complete nutrient media (sulfur sufficiency) contained 2.5 mM Ca(NO_3_)_2_*4H_2_O, 2.5 mM KNO_3_, 0.5 mM KH_2_PO_4_, 2 mM Na_2_SiO_3_*9H_2_O, 1.0 mM MgSO_4_*7H_2_O, 46.26 µM H_3_BO_3_, 9.15 µM MnCl_2_*4H_2_O, 0.76 µM ZnCl_2_, 0.32 µM CuCl_2_*2H_2_O, 0.016 µM (NH_4_)_6_Mo_7_O_24_*4H_2_O, 0.10 µM CoCl_2_*2H_2_O, and 62.5 µM FeCl_2_*4H_2_O, provided as Fe-EDTA chelate. The sulfur deficiency treatment corresponding to 12.5 µM SO_4_^2−^, was obtained by supplementing the MgSO_4_*7H_2_O with MgCl_2_*6H_2_O. The nutrient solution was renewed once every three days, and containers were daily refilled with ultra-pure water to compensate for evapotranspiration losses.

Plants were sampled 27 days after imbibition, at the vegetative/tillering stage reaching 1–3 tillers, i.e. relative to developmental stages 2.10 to 2.30 in *S. viridis*, and stages 2.21 to 2.23 in *O. sativa*. For biochemical and molecular analysis, including targeted metabolite profiling, and RNA-sequencing, three bulked biological replicates were utilized, each one consisting of a composite sample originating from tissue (shoot or root) of three individual plants grown in separate hydroponics units. Each plant was sampled for root and shoot tissues, generating aliquots of i) a homogeneous pool from the mid-section of the root system, and ii) a transverse segment from the mid-section of the leaf blade, from the youngest fully expanded leaf from the main plant (Fig. 1A). The collected sample aliquots were immediately frozen in liquid nitrogen and stored at -80ºC until further processing.

### Determination of total sulfur and S-containing metabolites

The content of total sulfur was analyzed in the shoots and roots using an inductively coupled plasma mass spectrometry (ICP-MS), as described by Almario et al. [58]. The plant material was dried at 60ºC for 48 h, and approximately 25 mg of homogenised samples were digested in metal-free polypropylene centrifuge tubes by 0.5 ml concentrated nitric acid (68%) overnight at room temperature, followed by digestion at 100 ºC for 40 min. After mineralization, the samples were diluted to approximately 5.0 ml with ultra-pure (deionized) water and centrifuged at 4 °C at 2000 g for 30 min. The elemental analysis was performed using an Agilent 7700 ICP-MS (Agilent Technologies, Santa Clara, California, USA) in the standard mode of operation. Sulfate was measured using ion chromatography as described in Dietzen et al. [10]. Approximately 50 mg of shoot or root samples were extracted by 0.5 ml of sterile ultra-pure water under constant shaking at 1500 rpm for 1 h at 4 ºC, and subsequent heating to 95 °C for 15 min. After centrifugation at 12700 rpm for 25 min at 4 ºC the supernatants were diluted 1:4 with sterile ultra-pure water prior to injection into the ion chromatograph (Dionex Aquion ICS-1100 chromatography system, Thermo Scientific, Waltham, Massachusetts, USA). Sulfate was separated on a Dionex IonPac AS22 4 × 250 mm analytical column, (Thermo Scientific™), using 4.5 mM Na_2_CO_3_ / 1.4 mM NaHCO_3_ as eluent at a flow rate of 1.2 ml min^−1.^ Sulfate was quantified using four standards (0.25 mM, 0.5 mM, 1 mM, and 2 mM K_2_SO_4_). Cysteine and glutathione were quantified as their monobromobimane derivatized products, following a modified protocol of the method described by Anoman et al. [59]. Approximately 50 mg of frozen plant material were cryogenic-pulverized in an agitator ball mill using glass beads, and 0.5 ml of 0.1 M HCl was added to each sample, followed by incubation under constant shaking at 1500 rpm for 40 min at 4 ºC. After centrifugation at 12700 rpm for 25 min at 4 °C, 60 μl of the supernatant was neutralized with 100 μl of 0.25 M CHES-NaOH (pH 9.4), and disulfides were reduced with 35 μl of freshly prepared 10 mM dithiothreitol. The reaction was incubated for 40 min at 22 °C. Five μl 25 mM monobromobimane were added and the samples were incubated for 15 min at 22 °C in the dark. To stop the reaction, 110 μl 100 mM methanesulfonic acid was added, followed by centrifugation at 12700 rpm for 30 min at 4 ºC. The derivatized thiols were separated by reverse-phase high-performance liquid chromatography (UltiMate™ 3000, Thermo Scientific). Forty μl of the samples were injected into a C18 HPLC column (EurospherⓇ 100–3 C18 ODS, C18 150 × 4 mm, 5 μm particle size, Knauer, Berlin, Germany) and eluted in a linear gradient from 4 to 20% eluent A (90% methanol, 0.25% acetic acid, pH 3.9) in eluent B (10% methanol, 0.25% acetic acid, pH 3.9), with a constant flow rate of 1 ml/min. MBB-conjugates were detected fluorimetrically at 470 nm after excitation at 390 nm with an FLD-3100 Fluorescence Detector.

### RNA isolation and quantitative RT-PCR

Total RNA was isolated with PureLink™ Plant RNA Reagent (Invitrogen, Thermo Scientific™, Waltham, Massachusetts, USA), using the small-scale isolation procedure recommended by the supplier, with minor modifications. Approximately 50 mg of shoot or root tissue samples were cryogenic-pulverized and kept frozen in liquid nitrogen before extraction. Each sample was resuspended by brief vortexing with 0.5 ml of pre-cold (4 °C) PureLink™ Plant RNA Reagent and incubated at room temperature for 5 min in a vertical rotating shaker. After centrifugation at 12700 rpm for 2 min at room temperature, the supernatant was transferred to a new RNase-free microtube containing 0.1 ml of 5 M NaCl, and 0.3 ml of 24:1 (v/v) chloroform-isoamyl alcohol was added, thoroughly mixed, and centrifuged at 12700 rpm for 10 min at 4 °C. The aqueous phase was recovered into a new microtube with an equal volume of 4 M LiCl-isopropyl alcohol (3:1 v/v) and incubated overnight at -20 °C. On the following day, the mixture was centrifuged at 12,700 rpm for 30 min at 4 °C, and the supernatant was decanted and replaced by 1.0 ml of 75% ethanol. Finally, the solution was centrifuged at 12700 rpm for 5 min at room temperature, the supernatant was completely discarded, and the microtubes were left open in the fume hood for 5 min to air-dry. The isolated RNA was resuspended in 20 µl RNase-free water, and concentration was estimated spectrophotometrically using a nanodrop (NanoDrop™ 2000c Spectrophotometer, Thermo Scientific).

Reverse transcription and qPCR were performed exactly as previously described [10] using QuantiTect® Reverse Transcription kit and SYBR Green in a CFX96 Touch™ Real-Time PCR Detection System (Bio Rad, Munich, Germany). Primers used are listed in Supplemental Table S10 and the transcript levels were normalized to ubiquitin using the 2^−ΔΔCT^ method. All transcript levels quantified by qPCR had 4 biological replicates. Real-time q-PCR primers for *S.viridis* were designed with the *qprimerdb* tool (https://biodb.swu.edu.cn/qprimerdb), and primers for *O.sativa kitaake* with https://cgm.sjtu.edu.cn/3kricedb/ or using the CDS from Phytosome (*O.sativa kitaake V3.1*)*.*

For RNA sequencing the RNA samples were further purified with TURBO DNA-free™ Kit (Invitrogen, Thermo Scientific), in an adapted version of the routine DNAse treatment specified by the manufacturer. In a 0.5 ml microtube, a total amount of 2 µg of RNA was resuspended in 20 µl RNase-free water and gently mixed with 2.1 µl 10X TURBO DNase™ Buffer and 1.5 µl of TURBO DNase™ Enzyme. The reaction was incubated at 37 °C, and after 30 min the enzyme activity was stopped by the addition of 4.5 µl DNase Inactivation Reagent, followed by vortexing, and incubation for 5 min at room temperature. The suspension was centrifuged at 12700 rpm for 1.5 min at room temperature, and the supernatant containing the treated RNA was transferred to a new RNase-free microtube.

### Library preparation, RNA sequencing, and data processing and analysis

Quality control of RNA samples was performed before sequencing using the Bioanalyzer system (2100 Bioanalyzer Instrument, Agilent Technologies, Santa Clara, California, USA), and the RNA 6000 Nano Kit (Agilent Technologies, Santa Clara, California, USA), adopting a RIN cutoff value of at least 7 (2 μg; 50–200 ng/μl; OD260/280 = 1.8–2.1; OD260/230 > 1.5). The library preparation and RNA sequencing were performed by Novogene (Novogene Co., Ltd., Cambridge, UK). After the quality control procedures, a non-strand-specific RNA library construction was carried out using poly-T mRNA enrichment. The resulting mRNA was randomly fragmented and used for the first cDNA synthesis via random hexamer priming, after which the second-strand synthesis was accomplished based on Illumina specifications, with the addition of the buffer containing dNTPs, RNase H, and DNA polymerase I (Novogene Co., Ltd., Cambridge, UK). End repair and dA-tailing were performed prior to the ligation of adapter sequences. After insert size selection and PCR amplification, the ready library was fluorometrically quantified with Qubit™ (Thermo Scientific™, Waltham, Massachusetts, USA) as well as by RT-qPCR, and re-assessed with the Bioanalyzer system for size distribution. Libraries were sequenced using Illumina NovaSeq™ 6000 Sequencing System (Illumina, Inc., San Diego, California, USA), to produce paired-end 150 bp reads and coverage of 30–40 Mio reads per sample.

Output data containing the paired-end sequence data of each sample (duplicate files) and their corresponding associated FastQC quality reports were stored as FastQ (fq.gz) files. Additionally, a quality control step was performed using MultiQC version 1.11 [60] to check the raw data from sequenced libraries, including the total number of sequences and estimate duplicated sequence counts. When necessary, residual rRNA sequences were filtered-out with SortMeRNA version 4.3.4 [61], and then Trimmomatic version 0.39 [62] was used to remove the adapters employed in the sequencing. Low-quality sequences were trimmed using the ‘maxinfo’ method with a target length of 125 bp, and subsequently, MultiQC was used for a second quality control, and processability of over 90% was confirmed for all samples. HISAT2 version 2.1.0 [22] was used to align the sequenced fragments against the reference genome datasets from *O. sativa ssp. japonica* cv. Kitaake (“OsativaKitaake_499_v3.0.fa”) and *S. viridis* (“Sviridis_500_v2.0.fa”) FASTA files, both obtained from the Joint Genome Institute via Phytozome portal [21]. Read summarization was performed with featureCounts version 2.0.0 [23] and the resulting count matrix was used for downstream analysis.

The stringent differential expression analysis was performed with DESeq2 [24] and limma [25]. Here, we used log2 fold change > 1.5 or log2 fold change < -1.5, adjusted *P*-value < 0.01 cut off to obtain the DEGs, and then used the intersect of both analyses. To find the DEGs in the roots and shoots separately we used only results obtained with DESeq2 [24] with adjusted *P*-value < 0.01. Z-score was computed on a gene-by-gene basis by subtracting the mean and then dividing by the standard deviation. The obtained Z score was further used to create the heatmaps. Clustering was performed using the MeV software (http://mev.tm4.org/). Network analysis was performed in VirtualPlant (http://virtualplant.bio.nyu.edu/cgi-bin/vpweb/; [29], and multivariate networks between the DEGs and metabolites were created in using the significant (*P*-value < 0.05) pair-wise correlations in Cytoscape [63]. The Pearson correlation analysis was performed using the ‘Hmisc’ and ‘corrplot’ packages in R (https://www.R-project.org). Gene ontology (GO) enrichment analysis was performed in Biomaps app in VirtualPlant [29].

### Metabolic analysis

Plant metabolites were determined according to Fiehn et al. [64]. Metabolites were extracted from 35–40 mg of frozen plant material with 1.5 ml of a pre-cooled (4 °C) single-phase solvent mixture of methanol/chloroform/water (2.5:1:1 v/v/v) containing 5 µM ribitol. Samples were vortexed for 20 s, placed in a vertical rotating shaker at 4 °C for 6 min, and centrifuged for at 12700 rpm for 2 min at 4 °C. The supernatant was transferred to a new tube and stored at -80 °C for further processing. An aliquot of 50 µl of the extract was dried using a speed vacuum concentrator and subjected to a two-step automatic sample derivatization. Initially, 10 µl of freshly made methoxyamine hydrochloride 20 mg ml^−1^ in pyridine were added and the mixture was shaken for 90 min at 37 °C. Next, 90 µl N-Methyl-N-(trimethylsilyl)trifluoroacetamide (MSTFA) was added and the samples were shaken 30 min at 37 °C. After subsequent incubation for 2 h at room temperature, 1 µl of the derivatized compounds were analyzed by gas chromatography coupled to mass spectrometry as described [65]. MassHunter Workstation Qualitative Analysis Software (version B.06.00, Agilent Technologies, Santa Clara, California, USA) was used for metabolite identification by comparison of spectra to the NIST14 Mass Spectral Library (https://www.nist.gov/srd/nist-standard-reference-database-1a-v14), while a standard mixture containing all target compounds at a concentration of 5 µM was processed in parallel to the samples as a response check and retention time reference. Peaks were integrated using MassHunter Workstation Quantitative Analysis Software (version B.08.00, Agilent Technologies, Santa Clara, California, USA), and relative quantification was computed from the integration of metabolite peak areas normalized to the corresponding sample fresh weight used for extraction and the peak area of the internal standard (ribitol). Metabolic pathway analysis was done in MetGenMAP tool (http://bioinfo.bti.cornell.edu/cgi-bin/MetGenMAP) [66] and MapMan [67]. Metabolic pathway diagrams were downloaded from https://plantcyc.org, and DEGs were added accordingly.

### Supplementary Information

**Additional file 1:** **Supplemental Figure 1.** Sulfur deficiency in *O. sativa* and *S. viridis*. **Supplemental Figure 2.** Comparison of transcript levels under S deficiency as determined by RNA-Seq and RT-qPCR. **Supplemental Figure 3.** Mapman overview of DEGs in metabolic pathways in *O. sativa* and *S. viridis* under S-deficiency. **Supplemental Figure 4.** Pathway analysis of DEGs under S-deficiency in *O. sativa* and *S. viridis*. **Supplemental Figure 5.** Functional GO biological enrichment analysis in shoots and roots of *O. sativa* under S deficiency. **Supplemental Figure 6.** Functional GO biological enrichment analysis in shoots and roots of *S. viridis* under S deficiency. **Supplemental Figure 7.** Intersect of enrichment of GO biological terms in shoots and roots of *O. sativa* and *S. viridis* under S deficiency [68]. **Supplemental Figure 8.** Network analysis of the DEGs and metabolites in *O. sativa* and *S. viridis* under S-deficiency.

**Additional file 2:** **Supplemental Table 1.** DEGs in *O.sativa* (C_3_) under S-deficiency. **Supplemental Table 2.** DEGs in *S.viridis* (C_4_) under S-deficiency. **Supplemental Table 3.** Common DEGs under S-deficiency in Arabidopsis thaliana (*Maruyama-Nakashita et al., 2006; **Dietzen et al*., 2020)* and ***present study. **Supplemental Table 4.** GO terms enriched in upregulated DEGs for *O.sativa* (C_3_) under S-deficiency. **Supplemental Table 5.** GO terms enriched in upregulated DEGs for *S.viridis* (C_4_) under S-deficiency. **Supplemental Table 6.** GO terms enriched in downregulated DEGs for O.sativa (C_3_) under S-deficiency. **Supplemental Table 7.** GO terms enriched in downregulated DEGs for *S.viridis* (C_4_) under S-deficiency. **Supplemental Table 8.** GO terms for sub-networks in *O. sativa* (C_3_) under S-deficiency. **Supplemental Table 9.** GO terms for sub-networks in *S.viridis* (C_4_) under S-deficiency. **Supplemental Table 10.** RT-qPCR primers used in this study.

## Data Availability

The RNAseq datasets generated and analysed during the current study are available in the NCBI SRA repository, under a BioProject ID PRJNA1068798.
