# Supplementary material for: Transcriptional and metabolic profiling of sulfur starvation response in two monocots
Source: BMC Plant Biol. 2024 Apr 9;24:257. doi: 10.1186/s12870-024-04948-2 (PMC11003109; doi:10.1186/s12870-024-04948-2)
Supplement: Supplementary file 1 — Additional file 1: Supplemental Figure 1. Sulfur deficiency in O. sativa and S. viridis. Supplemental Figure 2. Comparison of transcript levels under S deficiency as determined by RNA-Seq and RT-qPCR. Supplemental Figure 3. Mapman overview of DEGs in metabolic pathways in O. sativa and S. viridis under S-deficiency. Supplemental Figure 4. Pathway analysis of DEGs under S-deficiency in O. sativa and S. viridis. Supplemental Figure 5. Functional GO biological enrichment analysis in shoots and roots of O. sativa under S deficiency. Supplemental Figure 6. Functional GO biological enrichment analysis in shoots and roots of S. viridis under S deficiency. Supplemental Figure 7. Intersect of enrichment of GO biological terms in shoots and roots of O. sativa and S. viridis under S deficiency [68]. Supplemental Figure 8. Network analysis of the DEGs and metabolites in O. sativa and S. viridis under S-deficiency. [file 12870_2024_4948_MOESM1_ESM.pdf]

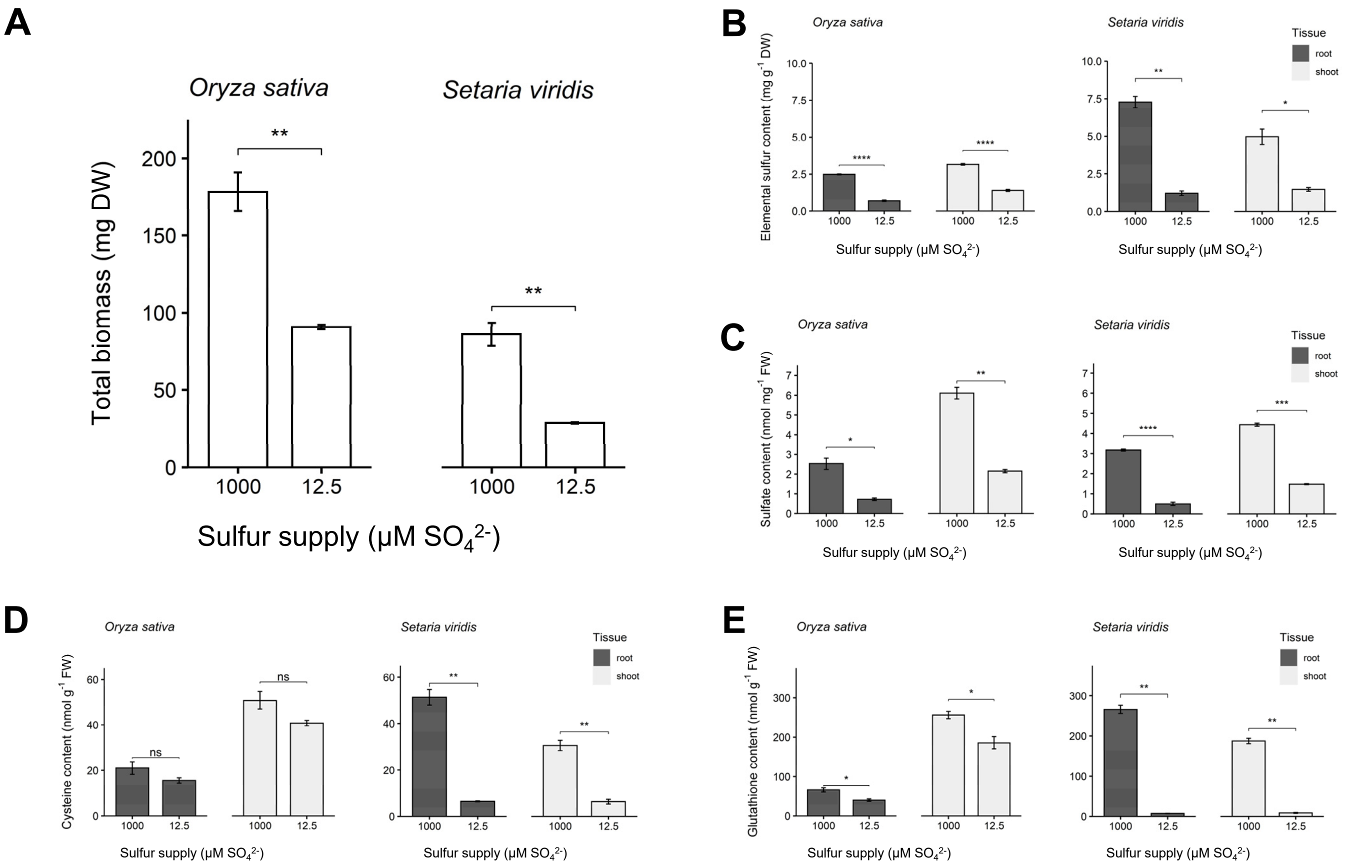

Supplemental Figure 1. Sulfur deficiency in *O. sativa* and *S. viridis*. A) Plant growth represented by total biomass accumulation in milligrams of dry weight (mg DW). **B)** Elemental sulfur, **C)** sulfate, **D)** cysteine, and **E)** glutathione accumulation in root ("dark-grey") and shoot ("light-grey") tissues. Plants were grown hydroponically under sulfur sufficiency (1000 μM SO_4^{2-}) and sulfur deficiency (12.5 μM SO_4^{2-}), for 20 days. Asterisks indicate statistically significant differences within the particular tissue of each species, according to t-test statistics with P＜0.05 (n = 3).

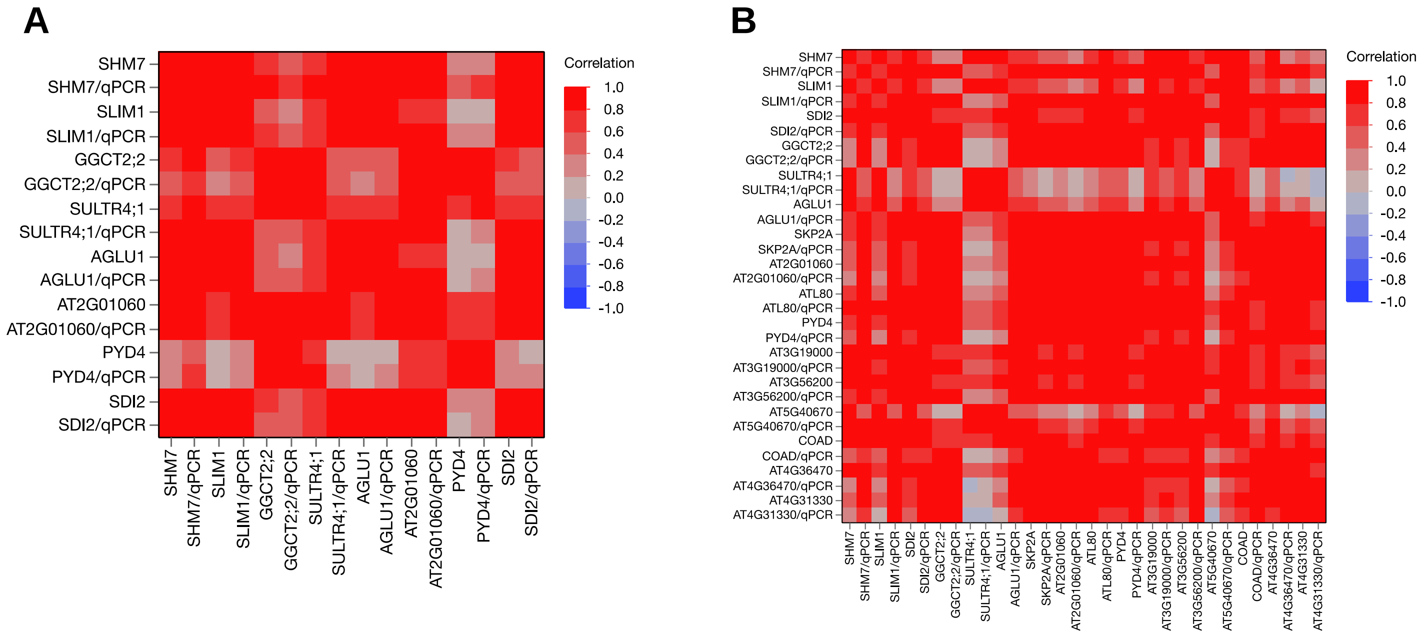

Supplemental Figure 2. Comparison of transcript levels under S deficiency as determined by RNA-Seq and RT-qPCR. A Correlation heat-map of fold change (lowS/C) of transcripts determined by RT-qPCR and RNA-Seq in *O. sativa*. **B** Correlation heat-map of fold change (lowS/C) of transcripts determined by RT-qPCR and RNA-Seq in *S. viridis*. Transcript levels were quantified by qPCR and normalized to Ubq with 4 biological replicates, or determined by RNAseq (TPM values).

A

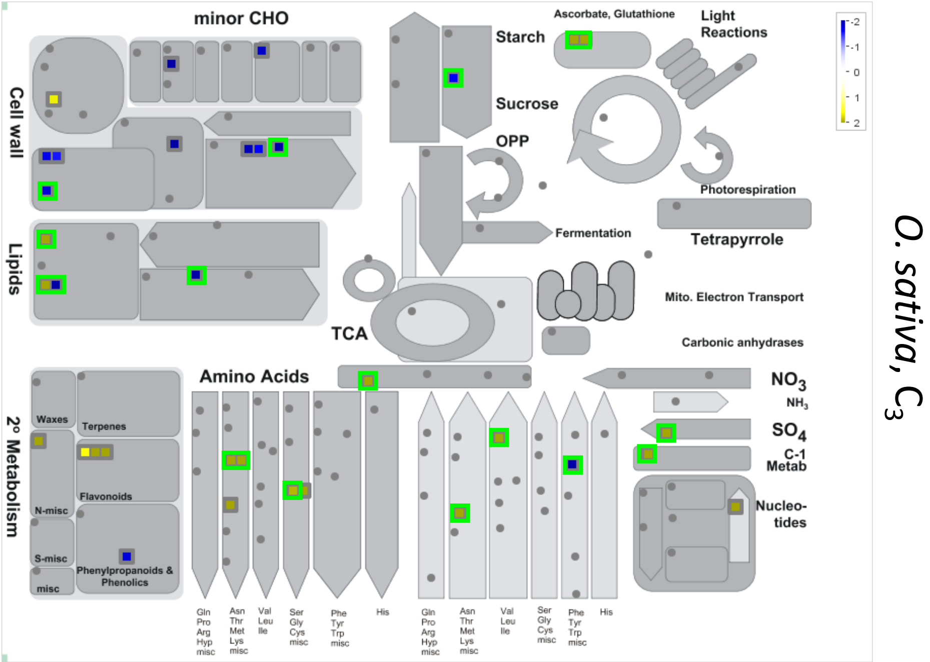

B

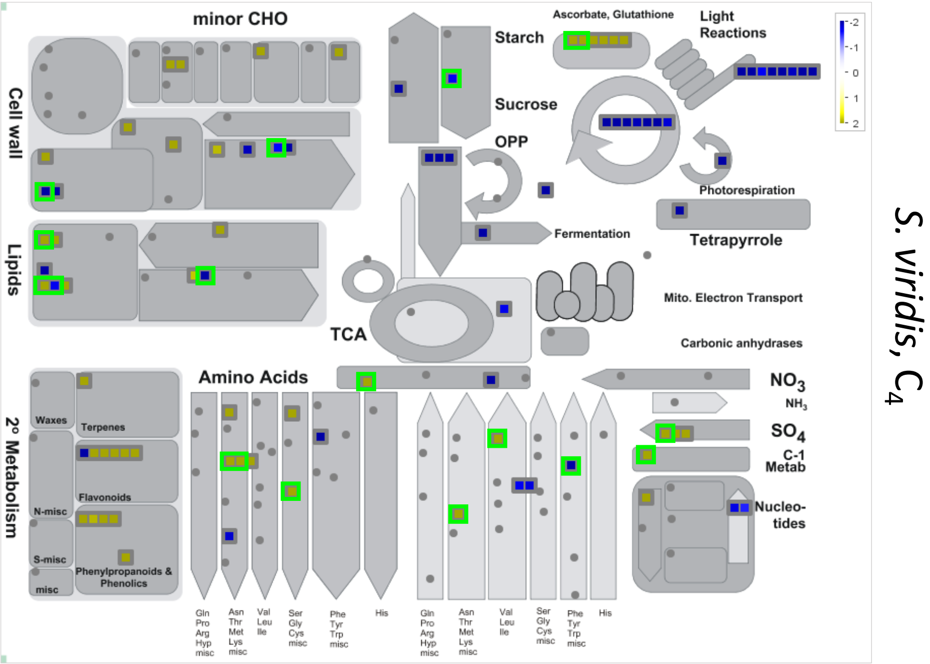

Supplemental Figure 3. Mapman overview of DEGs in metabolic pathways in *O. sativa* and *S. viridis* under S-deficiency. DEGs for S-deficiency in *O. sativa* **(A)** and *S.viridis* **(B)** were mapped onto the metabolic pathways represented in MapMan [67]. Shared DEGs in both *O. sativa* and *S. viridis* are highlighted in green.

A

	Number	Pathway name	p value
***O.sativa* Up-regulated genes**			
	1	S-methylmethionine cycle	0.000591
	2	superpathway of lysine, threonine and methionine biosynthesis II	0.002846
	3	methionine biosynthesis II	0.005191
*	4	cysteine biosynthesis I	0.008432
	5	methionine degradation II	0.010234
	6	4-aminobutyrate degradation I	0.020368
	7	proanthocyanidin biosynthesis from flavanols	0.020368
*	8	quercetinsulphates biosynthesis	0.020368
*	9	hydroxycinnamic acid tyramine amides biosynthesis	0.020368
*	10	GA12 biosynthesis	0.030403
*	11	leucopelargonidin and leucocyanidin biosynthesis	0.038812
*	12	leucodelphinidin biosynthesis	0.038812
***S.viridis* Up-regulated genes**			
	Number	Pathway name	p value
*	1	sulfate activation for sulfonation	0.000582
	2	methionine biosynthesis II	0.001635
	3	S-methylmethionine cycle	0.002993
*	4	sulfate reduction II (assimilatory)	0.013171
*	5	beta-caryophyllene biosynthesis	0.022905
	6	methionine degradation II	0.022905
	7	superpathway of lysine, threonine and methionine biosynthesis II	0.027123
*	8	acyl-ACP thioesterase pathway	0.045295
	9	4-aminobutyrate degradation I	0.045295
*	10	glutathione biosynthesis	0.045295
	11	proanthocyanidin biosynthesis from flavanols	0.045295

B

	Number	Pathway name	p value
***O.sativa* Down-regulated genes**			
*	1	hydroxyjasmonate sulfate biosynthesis	0.005361
	2	4-hydroxyphenylpyruvate biosynthesis	0.005361
*	3	quercetinsulphates biosynthesis	0.010695
*	4	ammonium transport	0.010695
*	5	tyrosine degradation I	0.021286
*	6	nicotianamine biosynthesis	0.021286
*	7	phenylalanine degradation III	0.03698
**S.viridis Down-regulated genes**			
	Number	Pathway name	p value
*	1	oxygenic photosynthesis	2.25E-06
*	2	Calvin-Benson-Bassham cycle	3.61E-06
*	3	glycolysis I	0.000544
*	4	glycolysis IV (plant cytosol)	0.000544
*	5	gluconeogenesis	0.000843
*	6	superpathway of cytosolic glycolysis (plants), pyruvate dehydrogenase and TCA cycle	0.00156
*	7	sucrose degradation to ethanol and lactate (anaerobic)	0.002319
*	8	superpathway of starch degradation to pyruvate	0.003088
*	9	superpathway of sucrose degradation to pyruvate	0.007176
*	10	leucine degradation I	0.011897
	11	4-hydroxyphenylpyruvate biosynthesis	0.013158
*	12	allantoin degradation to glyoxylate II	0.026149
*	13	allantoin degradation to ureidoglycolate II (ammonia producing)	0.026149
*	14	sulfolipid biosynthesis	0.026149
*	15	photorespiration	0.051086

Supplemental Figure 4. Pathway analysis of DEGs under S-deficiency in *O. sativa* and *S. viridis*. A Pathway analysis results for up-regulated DEGs under S-deficiency in *O. sativa* and *S. viridis*; species specific pathways are marked by an asterisks (*). **B** Pathway analysis results for down-regulated DEGs under S-deficiency in *O. sativa* and *S. viridis*; species specific pathways are marked by an asterisks (*). P-values for enriched pathways were obtained with MetGenMAP tool [66].

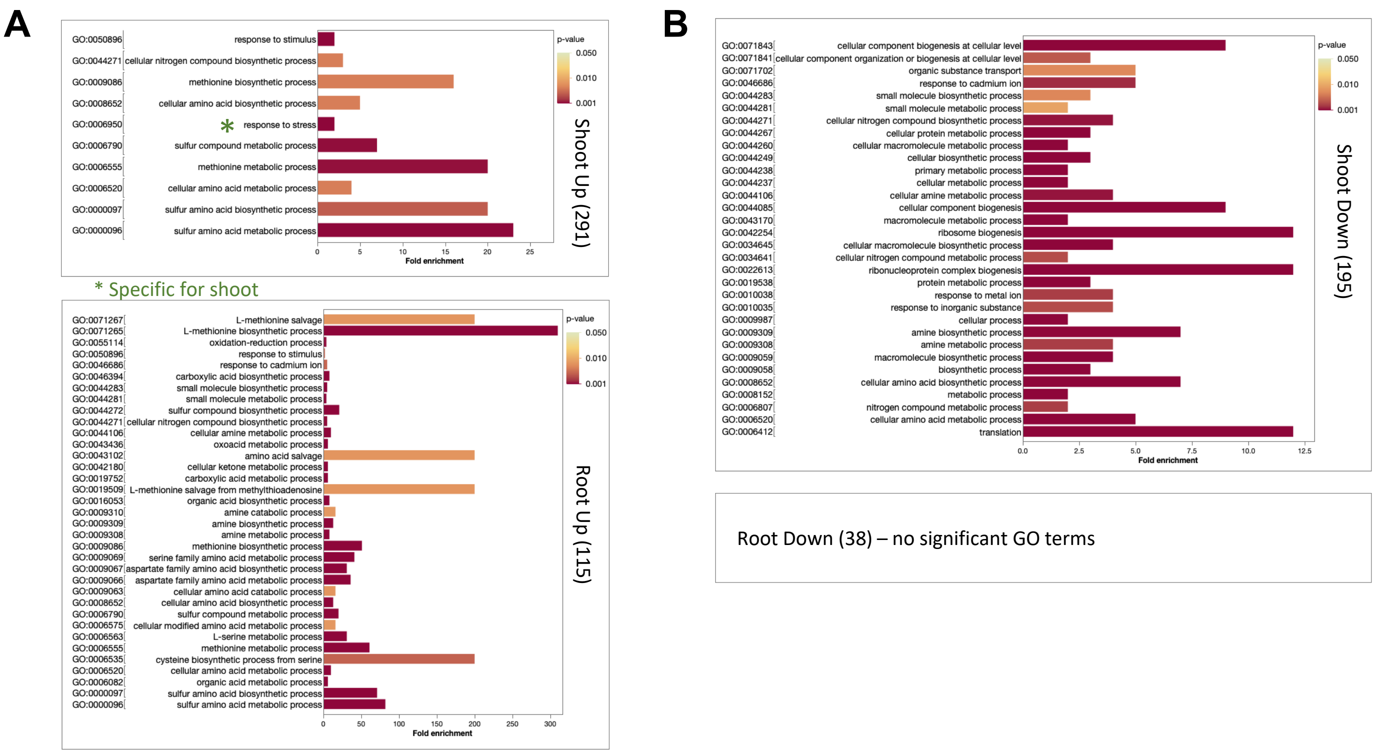

Supplemental Figure 5. Functional GO biological enrichment analysis in shoots and roots of *O. sativa* under S deficiency. A Functional GO biological enrichment analysis of up-regulated transcripts in *O. sativa* in shoots and roots. **B** Functional GO biological enrichment analysis of down-regulated transcripts in *O. sativa* in shoots and roots. Functional gene ontology (GO) enrichment analysis was performed with Biomaps app in VirtualPlant [29].

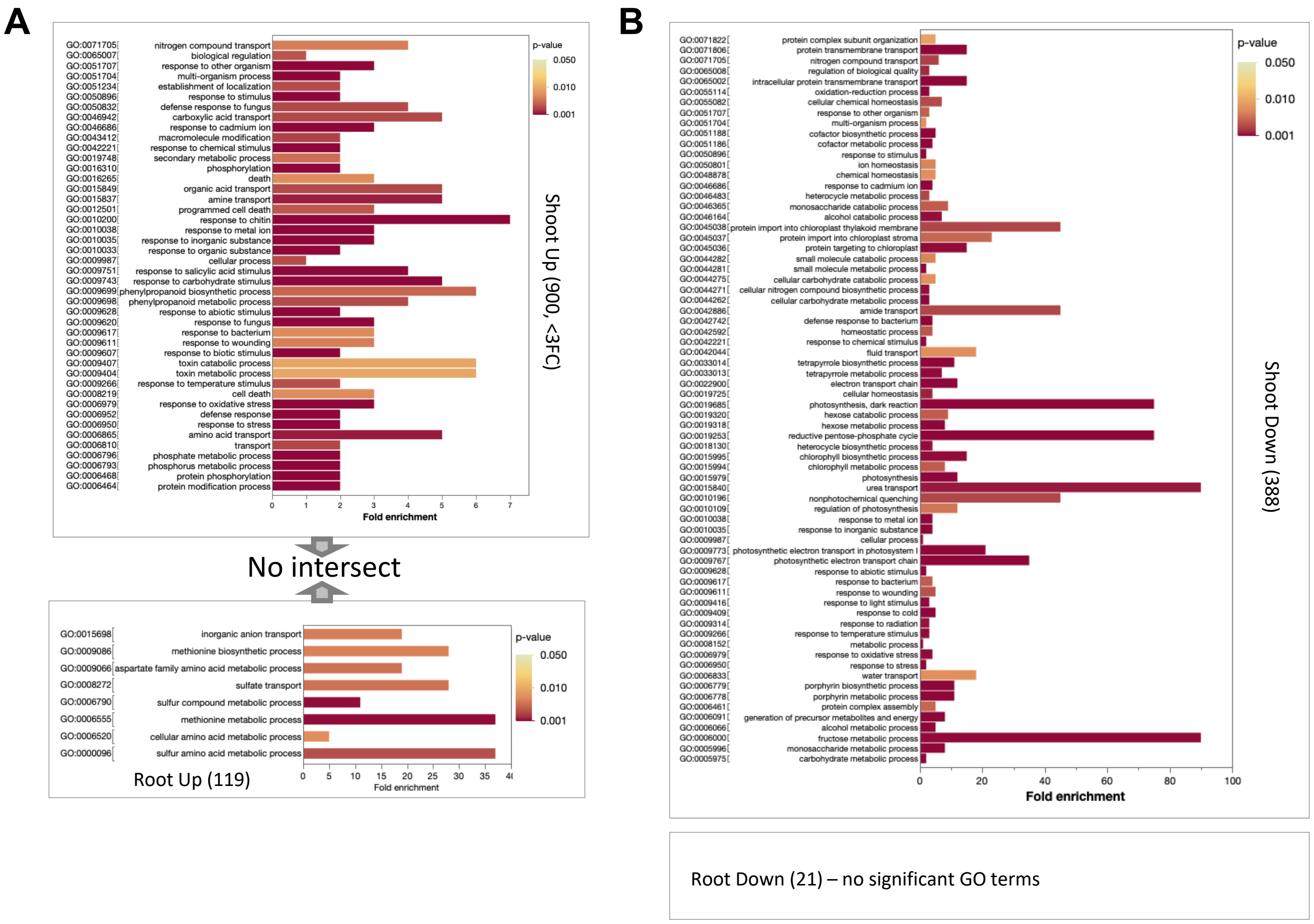

Supplemental Figure 6. Functional GO biological enrichment analysis in shoots and roots of *S. viridis* under S deficiency. A Functional GO biological enrichment analysis of up-regulated transcripts in *S. viridis* in shoots and roots. **B** Functional GO biological enrichment analysis of down-regulated transcripts in *S. viridis* in shoots and roots. Functional gene ontology (GO) enrichment analysis was performed with Biomaps app in VirtualPlant [29].

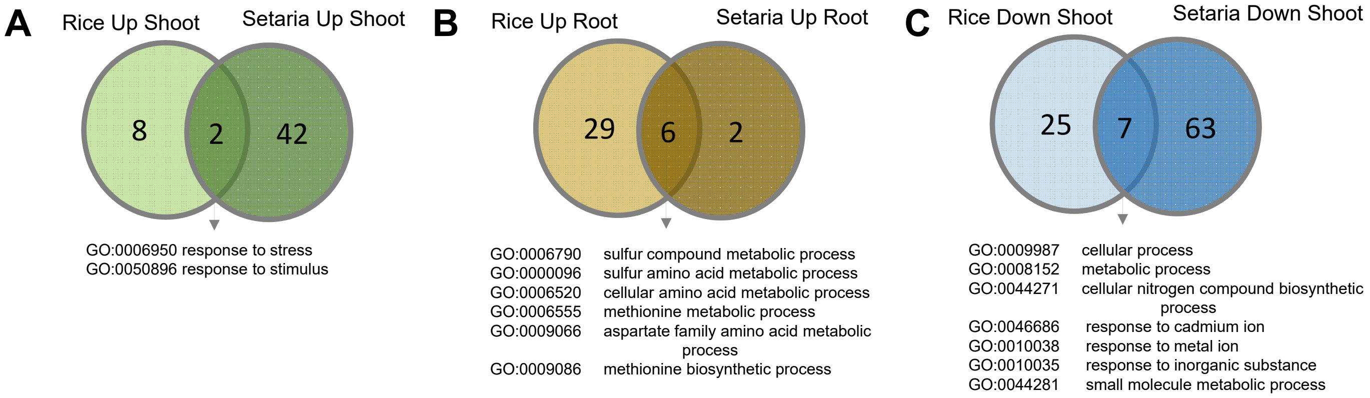

Supplemental Figure 7. Intersect of enrichment of GO biological terms in shoots and roots of *O. sativa* and *S. viridis* under S deficiency. A Intersect of functional GO biological enrichment analysis of up-regulated transcripts in *O. sativa* and *S. viridis* shoots. **B** Intersect of functional GO biological enrichment analysis of up-regulated transcripts in *O. sativa* and *S. viridis* roots. **C** Intersect of functional GO biological enrichment analysis of down-regulated transcripts in *O. sativa* and *S .viridis* shoots. Functional gene ontology (GO) enrichment analysis was performed with BINGO app [68] in Cytoscape [63].

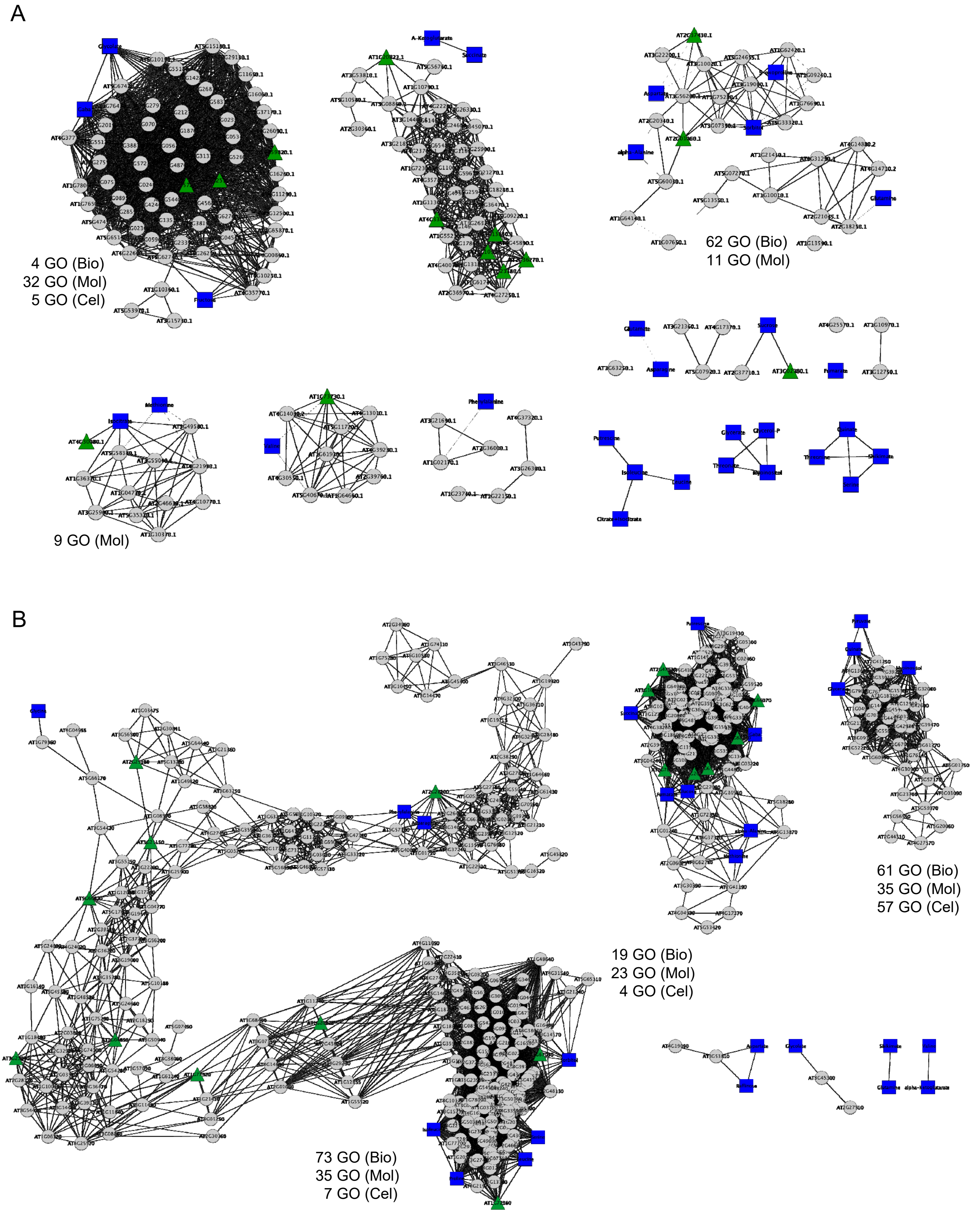

Supplemental Figure 8. Network analysis of the DEGs and metabolites in *O. sativa* and *S. viridis* under S-deficiency. A Pairwise correlations were used between all DEGs and 36 metabolites to generate the network in *O. sativa* (*p*-value ≤ 0.05). **B** Pair-wise correlations were used between all DEGs and 36 metabolites to generate the network in *S. viridis* (*p*-value ≤ 0.05). For each sub-network GO enrichment analysis was perform, and significant terms are displayed next to the corresponding sub-network, using Biomaps app in VirtualPlant [29]. Networks are visualized in Cytoscape, genes are represented in gray circles, metabolites in blue squares, and transcription factors in green triangles; edges width corresponds to level of correlation. Full list of GO terms listed in Supplemental Table 8 and 9.
